# Extend the Lifetime of Power Components in Series DC Motor Drives Using ANN-Based Adaptive Switching Frequency Optimization

**DOI:** 10.3390/s25226996

**Published:** 2025-11-16

**Authors:** Erkan Eren, Hakan Kaya, Salih Baris Ozturk

**Affiliations:** 1 Department of Electrical and Electronics Engineering, Zonguldak Bulent Ecevit University, Zonguldak 67100, Türkiye; e.eren.eee@gmail.com; 2 Department of Electrical Engineering, Istanbul Technical University, Istanbul 34469, Türkiye; ozturksb@itu.edu.tr

**Keywords:** battery-powered locomotive, insulated gate bipolar transistor (IGBT), mining, series DC motor, variable switching frequency, artificial neural network (ANN), embedded systems, machine learning, sensor-based signal processing, adaptive control algorithms, real-time decision making

## Abstract

This study presents an Artificial Neural Network (ANN)-based adaptive switching frequency control strategy for series Direct current (DC) motor drives used in battery-powered mining locomotives, aiming to extend the lifetime of critical power-electronic components such as Insulated Gate Bipolar Transistors (IGBTs) and DC bus capacitors. In embedded systems for electric traction, two dominant degradation factors, motor current ripple and IGBT temperature fluctuation, significantly shorten component lifetimes. Conventional fixed switching frequencies impose a trade off: higher frequencies reduce current ripple but increase IGBT losses and temperature, while lower frequencies yield the opposite effect. Consequently, an adaptive variable switching frequency control algorithm is proposed to perform real-time decision making by predicting the optimal switching frequency that minimizes both motor current ripple and IGBT thermal fluctuations. The proposed algorithm was trained with a dataset acquired from current sensors, NTC temperature sensors, and a potentiometer defining the target current (PWM duty). Performance comparisons with a fixed frequency demonstrate that the ANN-driven approach maintains an average current ripple of less than 5% (average) and 10% (maximum), while the lifetime of the IGBT and capacitors improves. A fairness index was defined to quantify the relative lifetime improvement of the IGBT and capacitor, revealing that the proposed variable frequency switching model enhances the overall system performance by up to 13 times compared to fixed-frequency operation. These results confirm that the integration of embedded machine learning and adaptive control algorithms can substantially enhance the durability and efficiency of power-electronic systems in real-time industrial applications.

## 1. Introduction

Among the sectors with the highest global energy demand, the transportation sector is the most prominent. As an alternative to petroleum-powered internal combustion engine vehicles, zero-emission battery electric vehicles (Electric Vehicles—EVs) have come to the forefront, offering great potential for decarbonizing the transportation sector [1]. Moreover, these vehicles provide safe, efficient, and environmentally friendly solutions in locations where the use of internal combustion engines poses a risk. In this context, battery-powered electric locomotives are also increasingly being used in underground mining operations as an alternative means of transportation. These locomotives are especially preferred in deep underground mines containing flammable gases such as methane, as they provide safe transport without emissions [1,2].

The main components of EVs include batteries, battery management systems, motors, inverters, and converters. Although Permanent Magnet Synchronous Motors (PMSMs) have been more widely adopted in recent years for electric vehicles, Series DC motors are still used in battery-powered locomotives deployed in mines due to their high torque, simple control structure, lower investment costs, and durability under harsh mining and dusty conditions. The use of PMSMs in mining environments can be costly due to the need for special cooling systems and materials to maintain performance. In such motors, a rise in the temperature of permanent magnets (PMs) reduces the magnetic field and consequently the torque generation. Under excessive heating, there is a risk of permanent demagnetization. Measuring the temperature of PMs directly is difficult because they are rotating components, requiring either cabling to moving parts or wireless systems, which present issues in terms of robustness and cost. Furthermore, if the stator winding temperature is not properly monitored, heat may transfer across the air gap to the rotor and damage the magnets. Therefore, accurate monitoring of stator temperature is critical for PMSMs [2,3,4,5,6]. In harsh environments such as underground mines, motor control systems must also withstand factors such as dust, humidity, and temperature fluctuations. Accordingly, the equipment and control methods must be suitable for such conditions. Due to these disadvantages of PMSM motors, although certain drawbacks arise from the use of mechanical commutators and brushes in series DC motors—such as periodic maintenance requirements and mechanical wear—series DC motors are still preferred over PMSM motors in mining applications.

In addition to efficiency, the reliability of the system and the lifetime of the materials used in electric vehicles are of paramount importance. Similarly to EVs, there are ongoing studies aimed at improving the efficiency of battery-powered locomotives used in mining, extending the lifetime of components, and reducing system failure rates [2]. In this context, the use of variable switching frequency in power converters (inverters and converters) has emerged as one of the most effective approaches to enhance system efficiency and extend component lifetime without increasing cost [3].

The IGBT, one of the key semiconductor devices in power electronics, is used in a wide range of fields, including transportation, industrial applications, lighting, consumer electronics, medical devices, and defense. Particularly in parallel with the increase in electric vehicle production, the use of IGBTs has increased. As a result, research and development on IGBT technologies and control techniques have intensified. Due to the limited energy source in EVs, minimizing the losses in IGBTs used in control units is essential to improve system efficiency. The losses in IGBTs are typically classified as conduction losses and switching losses. Conduction losses are static and are mostly dependent on the device technology. Switching losses, on the other hand, depend directly on the amplitude of the current passing through the IGBT and the switching frequency, assuming a constant DC bus voltage [3,4,5]. One method of reducing switching losses is to lower the switching frequency. However, this can lead to an increase in current ripple, which in turn causes a higher torque ripple under load conditions and negatively affects the passive and active components of the control unit [6,7,8,9]. Environmental loads such as humidity, mechanical vibration, and temperature are major factors affecting IGBT lifetime. Many studies have identified the junction temperature of the semiconductor devices as one of the most critical parameters impacting the lifetime of IGBT power modules. Different material layers with varying coefficients of thermal expansion in the IGBT module lead to mechanical stress, resulting in material fatigue and reduced device lifetime [10]. Generally, the IGBT lifespan is limited by the junction temperature, and unless it is adequately controlled, the risk of failure due to thermal cycling and overheating increases significantly. In [11], a simplified IGBT condition evaluation method using the operating interval segmentation (OIS) method to reduce simulation time while ensuring accuracy is proposed. It introduces a basic segmentation model, estimates temperature and stress trends, and enables the practical lifetime assessment of IGBTs, which is validated by test results. In addition, ref. [12] presented a study on real-time IGBT junction temperature estimation using NTC sensors and power loss calculations, in which the real-time junction temperature was estimated through power loss modeling combined with NTC sensor feedback to enhance inverter reliability. Their findings highlighted the direct correlation between accurate junction temperature estimation and improved lifetime prediction of IGBT modules. Furthermore, ref. [13] proposed a novel Remaining Useful Life (RUL) estimation approach based on a Volterra k-Nearest Neighbor Optimally Pruned Extreme Learning Machine (VKOPP) model, utilizing degradation data to predict the aging behavior of IGBTs with high accuracy. These studies emphasize that both real-time temperature estimation and data-driven lifetime prediction are crucial for enhancing the durability and reliability of IGBT-based power electronic systems.

In recent years, several studies have also employed machine learning and artificial intelligence techniques to predict and optimize the thermal and degradation behavior of power semiconductor devices. For instance, ref. [14] proposes an IGBT junction temperature estimation method that responds much faster than NTC sensors, offers high accuracy under dynamic conditions, and accounts for thermal coupling. In doing so, it aims to enhance the reliability and power density of traction inverters. Similarly, in [15], a CNN-LSTM (Convolutional Neural Network–Long Short-Term Memory) based prediction algorithm was developed to model and forecast the performance degradation of the gate oxide layer in IGBT devices. In another study, ref. [16] reviewed the application of neural networks in electrical drives, highlighting their potential in adaptive control, reliability estimation, and fault diagnosis of power converters. These machine learning–based approaches demonstrate that neural networks are effective tools for modeling the nonlinear thermal, diagnostic, and aging characteristics of IGBTs and electric drives, especially under dynamic operating conditions. Furthermore, beyond IGBT lifetime prediction, several studies have demonstrated the broader applicability of ANN and related intelligent strategies within power electronics and motor drive systems. For instance, ref. [17] proposed a fault-tolerant control scheme based on current space vectors to sustain operation under total sensor failures, illustrating how advanced control and diagnostic logic can enhance drive reliability. Likewise, ref. [18] ANN-based fault diagnosis system to detect electrical faults in asynchronous motors using vibration data. These studies reinforce the versatility of ANN-centric and intelligent control methodologies in power electronic applications, supporting their integration into adaptive control and condition-monitoring frameworks such as the one proposed in this work.

Pulse Width Modulation (PWM) drivers are used to operate motors at different speeds and load points. In practical scenarios, the current ripple in the motor significantly reduces motor life. This ripple can be minimized by increasing the PWM switching frequency. If the switching frequency is significantly higher than the motor’s electrical time constant (L/R), current ripple is further reduced [19]. The ripple in the DC bus current also shortens the lifetime of DC bus capacitors. Reducing the current ripple requires increasing the switching frequency. However, doing so also increases switching losses. Therefore, an optimal switching frequency must be determined to balance these opposing effects—reducing losses while minimizing ripple and extending capacitor life [3].

Studies in the literature have shown that increasing the switching frequency is an effective solution to mitigate the adverse effects of motor current ripple on both motor and power semiconductors, without increasing hardware costs [7,8,9,20]. Accordingly, adaptive methods such as variable gate resistance and variable switching frequency are employed. In one study, a variable switching frequency method was proposed for a two-level, three-phase inverter to reduce switching losses, and the current ripple was estimated using a complex mathematical model [6]. Another study used Fourier analysis to analytically calculate the fundamental and harmonic components of motor currents and proposed a control strategy to jointly optimize the switching frequency and motor current to reduce switching-related losses [21]. In a different work, a switching frequency scheme was developed based on motor current ripple and the switching frequency using mathematical analysis [5]. Yet another study implemented a variable switching frequency strategy to prevent the negative consequences of torque ripple caused by AC motor current ripple [22].

In this study, the use of variable switching frequency is proposed for battery-powered electric locomotives used in mining applications to maintain IGBT temperatures and series DC motor current ripple within acceptable limits, thereby improving the operational lifetime of both IGBTs and DC bus aluminum capacitors. Accordingly, the system performance was analyzed under various load scenarios. The performance of the locomotive was examined under continuously varying load conditions. The effect of switching frequency on motor current and IGBT temperature was observed during motor load variations. Under fixed switching frequency, it was noted that the IGBT temperatures increased rapidly, and the motor current ripple became more pronounced with changes in load. However, with the use of variable switching frequency, the system adapted better to these changes and fluctuations in both motor current and IGBT temperature were significantly minimized. Based on the data obtained from each scenario, a dataset was constructed, and an ANN model was developed. Using ANN, the optimal switching frequency was predicted based on IGBT temperature, motor current, and PWM duty cycle. During the training process, different combinations of IGBT temperatures, motor currents, and switching frequencies were fed into the ANN model, and optimal switching frequencies were estimated for varying load conditions. The analysis demonstrated that variable switching frequency control, combined with ANN-based optimization, significantly reduced both the motor current and the IGBT temperature ripple, thereby extending the lifespan of the system components and improving overall system efficiency.

### 1.1. Contributions

The main contributions of this paper are as follows:

- A novel control method is introduced that adaptively adjusts the switching frequency in real-time based on load conditions to reduce motor current ripple and IGBT temperature fluctuations.
- An ANN model is developed and trained using motor current, IGBT temperature, and PWM duty cycle as inputs to predict the optimal switching frequency for minimizing thermal fluctuations and motor current ripple. The training data for the ANN is primarily derived from theoretical modeling, as obtaining extensive field data is challenging in practice.
- A real-time, microcontroller-based algorithm is proposed to predict the optimal switching frequency.
- Compared to fixed-frequency methods, the ANN-based adaptive control significantly reduces current ripple (below 5%) and extends IGBT and capacitors lifetime enhancing overall system efficiency.

### 1.2. Organization

The remainder of this paper is organized as follows. In Section 2, a four-quadrant driver circuit employing a PWM-based PI controller that enables both directional and speed control of a series-wound DC motor is introduced. The electrical model of the series DC motor is presented, and the minimum, maximum, and ripple values of the motor current are mathematically formulated. In addition, the IGBT power losses—both conduction and switching—are calculated, and junction temperatures are used to estimate the semiconductor lifetime based on the LESIT model. Furthermore, the lifetime of the DC link capacitors is analytically evaluated using ambient conditions and manufacturer specifications.

In Section 3, the accuracy of the developed mathematical model is validated through computer-based simulation studies of the driver circuit. The simulation results are presented in comparison with the analytical expressions, and the consistency between them is assessed.

In Section 4, in order to validate the theoretical and simulation studies, field tests are conducted on battery-powered locomotives used in underground mining operations. A hardware prototype of a series DC motor driver circuit, compatible with the proposed control structure, is designed and implemented. During field tests, motor current values are measured under fixed PWM duty cycle and fixed current conditions at different switching frequencies, and these results are compared with both theoretical and simulation data. Finally, the IGBT temperatures are measured under field conditions, showing strong agreement with the theoretical predictions.

In Section 5, an algorithm is developed to determine the optimum variable switching frequency with the aim of reducing thermal cycling in IGBTs and minimizing peak current ripples in DC bus capacitors. At the hardware level, smooth frequency transitions are implemented, and the control algorithm is designed in a real-time, microcontroller-supported architecture. In the initial stage, frequency switching between 1 kHz and 10 kHz is performed based on threshold temperature values, and the results indicate a reduction in temperature fluctuations. However, as the threshold-based approach proves insufficient under varying load and temperature conditions, a neural network-based decision mechanism is proposed in Section 6 to determine the optimal frequency based on the observed data. In the proposed ANN model, the motor current, the PWM duty cycle, and the IGBT temperature are used as input parameters, and the output corresponds to the predicted optimum switching frequency. The implementation of the trained neural network in the real-time hardware control algorithm for determining the optimal frequency is explained in detail.

Section 7 analyzes the impact of the proposed ANN-based model on the driver circuit parameters and components. The proposed method is evaluated under multiple test scenarios involving various load and PWM duty cycles, as well as a real route profile. The results demonstrate the positive effects of the method on the operational lifetime of critical components such as IGBTs and capacitors.

Finally, the last Section 8 of the paper presents a general discussion, summarizes the key findings of the study, and outlines suggestions for future research directions.

## 2. Theoretical Modeling of a Series DC Motor Drive and Derivation of the Operational Lifetime Expressions for Power Electronic Components

**Figure 1:**
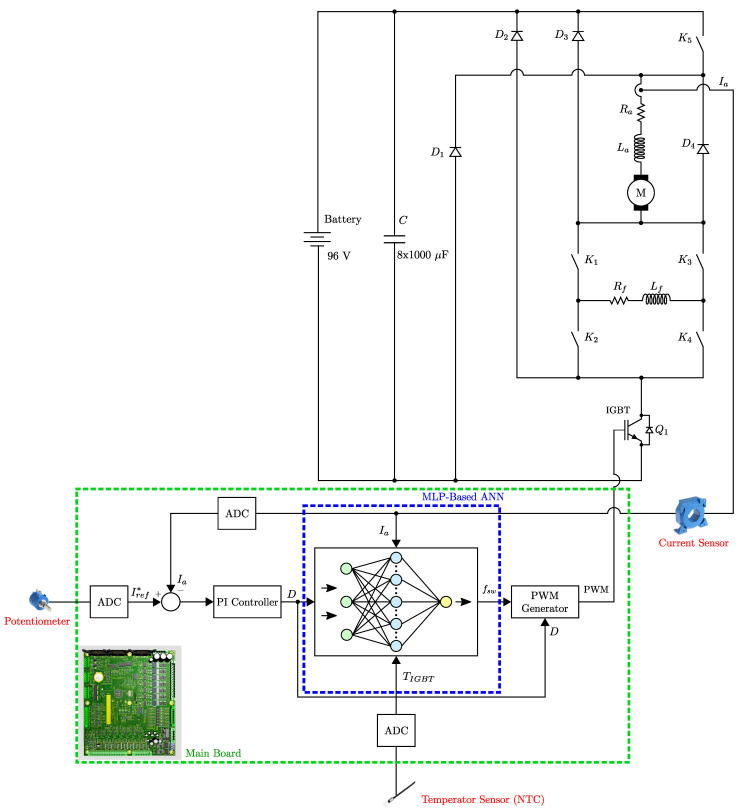
General block diagram of the designed system.

Figure 1 illustrates a four-quadrant driver designed to control both the speed and direction of a series-wound DC motor. Four-quadrant drivers are capable of operating in both forward and reverse directions, enabling battery-powered locomotives to move bidirectionally. This bidirectional capability supports operational requirements such as maneuvering and parking. Furthermore, it provides effective control at low speeds, enhancing locomotive performance during slow or precise movements. These drivers operate efficiently over a wide range of speeds and load conditions, making them highly suitable for various operational demands.

In the circuit shown in Figure 1, a 96 V battery serves as the power supply. A capacitor bank is connected in parallel to suppress voltage fluctuations caused by sudden load changes, serving as an energy buffer. The motor current is regulated based on a user-controlled analog signal. This signal is digitized using an Analog-to-Digital Converter (ADC), and then used to compute the reference current $I_{\mathrm{ref}}^{*}$ proportionally to the maximum value of the user-controlled signal and the system’s predefined maximum current.

Meanwhile, the actual motor (armature) current $I_{a}$ is measured during each PWM switching cycle using a LEM current transducer. The measured value is compared with the reference, and the error is processed by a proportional–integral (PI) controller. The integral term is limited within predefined bounds to ensure system stability and prevent wind-up. The PI controller output determines the duty cycle of the PWM signal. The motor’s average supply voltage is controlled via switching element $Q_{1}$ (an IGBT), thereby regulating the motor current. This control loop runs continuously, updating the PWM signal in real time based on the current tracking.

Diodes $D_{1}$ through $D_{4}$ are included to handle reverse voltages and feedback currents resulting from the inductive nature of the motor. The diodes $D_{2}$, $D_{3}$, and $D_{4}$ provide paths to safely discharge the energy stored in the windings when $Q_{1}$ turns off. The diode $D_{1}$, in conjunction with $Q_{1}$, manages the reverse current during regenerative braking.

In series-wound DC motors, direction control is achieved by reversing the current through either the armature or the field winding. In this circuit, the motor is modeled using armature resistance $R_{a}$, armature inductance $L_{a}$, field resistance $R_{f}$, and field inductance $L_{f}$. The field current direction is altered using four contactors—$K_{1}$ to $K_{4}$—which also assist during regenerative braking operations.

The mathematical model of series DC motors is expressed through both electrical and mechanical equations. In series-wound DC motors, both the field winding and the armature winding are supplied through the same circuit. This implies that the same current flows through both the armature and the field windings.

The electrical model of a series DC motor can be derived using Kirchhoff’s Voltage Law. The voltage equation for the motor armature circuit is formulated as follows:

(1) $$V_{cc}=I_{a}R_{a}+I_{a}R_{f}+E\;$$

which is given in [23,24]. Here, $V_{cc}$ is the voltage applied to the motor terminals (V), $R_{a}$ is the armature resistance (Ω), $R_{f}$ is the field resistance (Ω), and *E* is the back electromotive force (EMF) (V).

The back EMF is proportional to the motor speed and can be defined as

(2) $$E=k_{e}\cdot \phi \cdot \omega \;$$

where $k_{e}$ is the back EMF constant, ϕ is the magnetic flux, and ω is the electrical angular speed of the motor (rad/s). In series DC motors, the magnetic flux ϕ is directly proportional to the armature current, i.e., $\phi \propto I_{a}$. Although magnetic saturation may occur, in the initial modeling phase, the approximation $\phi =k_{f}\cdot I_{a}$ can be assumed, where $k_{f}$ is the magnetic flux constant.

For four-quadrant drivers, the maximum ($I_{a_{max}}$), minimum ($I_{a_{min}}$), and ripple ($I_{a_{rip}}$) values of the motor current are defined as follows [25,26,27]:

(3) $$\%I_{a_{rip}}=\frac{I_{a_{max}}-I_{a_{min}}}{I_{a}}\times 100$$

(4) $$I_{a}=\frac{I_{a_{max}}-I_{a_{min}}}{2}$$

(5) $$I_{a_{min}}=\frac{V_{cc}}{R_{a}+R_{f}}\cdot \frac{1-e^{\left(\frac{D}{f_{sw}\cdot \frac{L}{R_{a}+R_{f}}}\right)}}{1-e^{\left(\frac{1}{f_{sw}\cdot \frac{L}{R_{a}+R_{f}}}\right)}}-\frac{E}{R_{a}+R_{f}}$$

(6) $$I_{a_{max}}=\frac{V_{cc}}{R_{a}+R_{f}}\cdot \frac{1-e^{\left(-\frac{D}{f_{sw}\cdot \frac{L}{R_{a}+R_{f}}}\right)}}{1-e^{\left(-\frac{1}{f_{sw}\cdot \frac{L}{R_{a}+R_{f}}}\right)}}-\frac{E}{R_{a}+R_{f}}$$

where $\%I_{a_{rip}}$ is the current ripple in percent, $I_{a_{min}}$ and $I_{a_{max}}$ are the minimum and maximum armature currents in amperes, $f_{sw}$ is the PWM switching frequency in hertz, and *D* is the duty cycle of the PWM signal.

### 2.1. IGBT Losses and Lifetime Estimation

In power modules, conduction losses during current flow and switching losses due to turn-on and turn-off operations must be taken into account. The heat generated from these losses is dissipated from the IGBT modules through specialized cooling systems [28,29,30].

Leakage losses are typically negligible compared to conduction and switching losses and are difficult to evaluate. Therefore, only conduction and switching losses are analyzed and modeled in this study.

The total power loss in the IGBT can be defined as  [29,30,31,32,33,34,35,36,37,38,39,40]:

(7) $$P_{\mathrm{total},\;\mathrm{IGBT}}=P_{\mathrm{sw},\;\mathrm{IGBT}}+P_{\mathrm{cond},\;\mathrm{IGBT}}.\;$$

Here, $P_{\mathrm{total},\;\mathrm{IGBT}}$ represents the total power loss, $P_{\mathrm{sw},\;\mathrm{IGBT}}$ is the switching loss, and $P_{\mathrm{cond},\;\mathrm{IGBT}}$ is the conduction loss.

Conduction losses are calculated using the following equation.

(8) $$P_{\mathrm{cond},\;\mathrm{IGBT}}=I_{c}\cdot V_{\mathrm{CE}(\mathrm{sat})}\;$$

where $I_{c}$ is the collector current during conduction and $V_{\mathrm{CE}(\mathrm{sat})}$ is the saturation voltage drop across the IGBT.

The turn-on and turn-off switching losses of the IGBT are given by:

(9) $$E_{\mathrm{sw},\mathrm{on},\mathrm{IGBT}}=E_{\mathrm{on}}{\left(\frac{I_{c}}{I_{c,\mathrm{ref}}}\right)}^{K_{i}}{\left(\frac{V_{\mathrm{cc}}}{V_{\mathrm{cc},\mathrm{ref}}}\right)}^{K_{v}}\times \left[1+TC_{\mathrm{sw}}\left(T_{j}-T_{j,\mathrm{ref}}\right)\right]$$

(10) $$E_{\mathrm{sw},\mathrm{off},\mathrm{IGBT}}=E_{\mathrm{off}}{\left(\frac{I_{c}}{I_{c,\mathrm{ref}}}\right)}^{K_{i}}{\left(\frac{V_{\mathrm{cc}}}{V_{\mathrm{cc},\mathrm{ref}}}\right)}^{K_{v}}\times \left[1+TC_{\mathrm{sw}}\left(T_{j}-T_{j,\mathrm{ref}}\right)\right].$$

In these equations, $I_{c,\mathrm{ref}}$, $V_{\mathrm{cc},\mathrm{ref}}$, and $T_{j,\mathrm{ref}}$ are the reference test conditions. The parameter $K_{i}$ is the current dependency exponent (typically 0.5–0.6), $K_{v}$ is the voltage dependency exponent (typically 0.6), and $TC_{\mathrm{sw}}$ is the temperature coefficient for switching losses (typically 0.005–0.006). The quantities $E_{\mathrm{on}}$ and $E_{\mathrm{off}}$ are the turn-on and turn-off switching energies, respectively. These values are generally provided by manufacturers based on experimental characterization. Reverse recovery losses $E_{rr}$ of the anti-parallel freewheeling diode (FWD) can be neglected in this study [35].

Thus, the total switching loss is given by:

(11) $$P_{\mathrm{sw},\mathrm{IGBT}}=\left(E_{\mathrm{sw},\mathrm{on},\mathrm{IGBT}}+E_{\mathrm{sw},\mathrm{off},\mathrm{IGBT}}\right)\cdot f_{\mathrm{sw}}.$$

The junction temperature of the IGBT can be estimated using the thermal resistance model [10,30,41,42,43,44,45]:

(12) $$T_{j}=P_{\mathrm{total},\mathrm{IGBT}}\cdot \left(R_{{\mathrm{th}}_{j-c}}+R_{{\mathrm{th}}_{c-hs}}+R_{{\mathrm{th}}_{hs-a}}\right)+T_{a}.\;$$

Here, $R_{{\mathrm{th}}_{j-c}}$ is the thermal resistance from junction to case, $R_{{\mathrm{th}}_{c-hs}}$ from case to heatsink, $R_{{\mathrm{th}}_{hs-a}}$ from heatsink to ambient and $T_{a}$ is the ambient temperature.

The lifetime of a semiconductor device is significantly affected by the temperature change ($\Delta T_{J}$) and the average junction temperature ($T_{m}$). These parameters directly influence the number of thermal cycles ($N_{f}$) and hence the operational lifetime of the IGBT.

The LESIT model is commonly used for estimating the lifetime of IGBT modules. This model, incorporating the Arrhenius and Coffin-Manson degradation laws, is given by [10,30,41,42,43,44,45]:

(13) $$N_{f}=A\cdot {\left(\Delta T_{J}\right)}^{\alpha }\cdot \exp\left(\frac{E_{a}}{k_{B}T_{m}}\right).$$

In this expression, $N_{f}$ denotes the number of cycles to failure. *A* and α are experimentally determined constants based on material and packaging characteristics. $\Delta T_{J}$ is the junction temperature change, where a larger temperature fluctuation imposes a higher mechanical stress on the IGBT due to mismatches in thermal expansion coefficients. $E_{a}$ is the activation energy representing the thermal energy required to initiate the failure mechanisms. $T_{m}$ is the mean junction temperature, where elevated levels accelerate mechanisms such as solder fatigue. $k_{B}$ is the Boltzmann constant, with a value of $8.617\times 10^{-5}$ eV/K.

### 2.2. Lifetime Estimation of Capacitors

After calculating the temperature, current ripple and voltage-related factors, the operational lifetime $L_{X}$ of capacitors can be estimated using the rated lifetime $L_{0}$ provided in the manufacturer’s datasheet  [3,46,47,48].

(14) $$L_{X}=L_{0}\cdot 2^{\left(\frac{T_{0}-T_{a}}{10}\right)}\cdot K_{i}^{\left(\left[1-{\left(\frac{I_{a}}{I_{0}}\right)}^{2}\right]\cdot \frac{\Delta T_{0}}{10}\right)}\cdot {\left(\frac{U_{a}}{U_{r}}\right)}^{-n}.\;$$

In this equation, $K_{i}$ is the empirical safety factor; $T_{0}$ is the maximum operating temperature specified by the manufacturer; $T_{a}$ is the ambient temperature; $I_{0}$ denotes the rated ripple current at $T_{0}$. The parameter $\Delta T_{0}$ represents the temperature rise, which is typically taken as 5 K for 105 °C capacitors and 10 K for 85 °C capacitors. $U_{a}$ denotes the applied voltage in the actual circuit, while $U_{r}$ is the rated voltage provided by the manufacturer. *n* is the voltage stress coefficient that defines how the applied voltage influences the lifetime of the capacitor. It is determined experimentally by the manufacturer.

## 3. Simulation-Based Validation of the Derived Theoretical Expressions for the Series DC Motor

To validate the accuracy of the mathematical model of the series DC motors presented in Section 2, the motor control circuit shown in Figure 1 was implemented in MATLAB^®^ 2020a software, and the theoretical results were compared with the simulation results. Therefore, a test environment was prepared in which IGBT losses, junction temperatures, and motor current values could be computed and/or measured as functions of the switching frequency and duty cycle using both the mathematical model and simulation results. In this section, all calculations and measurements are performed under fixed switching frequency conditions only.

In the analyzes, the system with electrical specifications listed in Table 1, the series DC motor with parameters listed in Table 2, an anti-parallel diode, a IGBT module, a mechanical load, and measurement probes for current, voltage, and torque were utilized.

The following quantities were obtained for fixed switching frequencies of $f_{sw}=1$ kHz, 5 kHz, and 10 kHz: total IGBT power losses ($P_{\mathrm{total},\mathrm{IGBT}}$, W), IGBT switching losses ($P_{\mathrm{sw},\mathrm{IGBT}}$, W), IGBT conduction losses ($P_{\mathrm{cond},\mathrm{IGBT}}$, W), minimum motor current ($I_{c,\min}$, A), maximum motor current ($I_{c,\max}$, A), nominal motor current ($I_{c}$, A), and IGBT junction temperature ($T_{j}$, °C).

**Figure 2:**
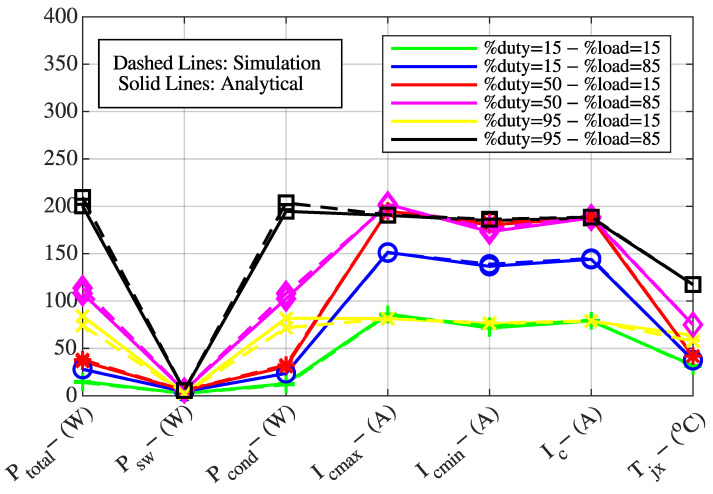
Comparison of theoretical and simulation results at a switching frequency of 1 kHz.

**Figure 3:**
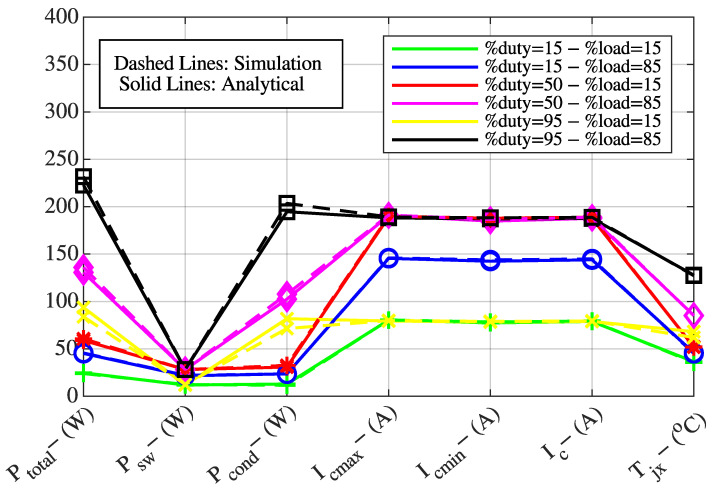
Comparison of theoretical and simulation results at a switching frequency of 5 kHz.

**Figure 4:**
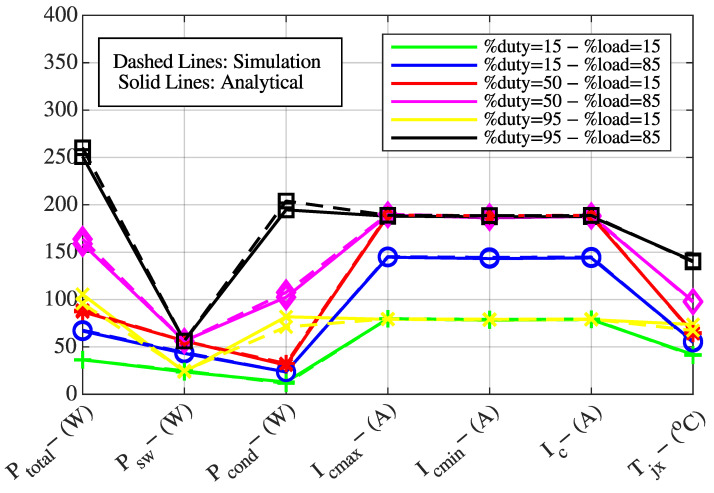
Comparison of theoretical and simulation results at a switching frequency of 10 kHz.

To verify the theoretical expressions presented in Section 2, the system was evaluated under load levels of 15% and 85%, and PWM duty cycles of 15%, 50%, and 95%. The theoretical results obtained for $f_{sw}=1$ kHz, 5 kHz, and 10 kHz are compared with the simulation results and presented in Figure 2, Figure 3 and Figure 4.

In Figure 2, Figure 3 and Figure 4, the Y-axis represents the value of the parameter given on the X-axis in terms of its corresponding unit indicated in parentheses. For example, total IGBT power losses are expressed in watts ($P_{\mathrm{total},\mathrm{IGBT}}$, W), maximum motor current in amperes ($I_{c,\max}$, A), and IGBT junction temperature in degrees Celsius ($T_{j}$, °C). For instance, in Figure 2, considering the blue curve, the values of total IGBT power losses ($P_{\mathrm{total},\mathrm{IGBT}}$, W), IGBT switching losses ($P_{\mathrm{sw},\mathrm{IGBT}}$, W), IGBT conduction losses ($P_{\mathrm{cond},\mathrm{IGBT}}$, W), maximum motor current ($I_{c,\max}$, A), minimum motor current ($I_{c,\min}$, A), nominal motor current ($I_{c}$, A), and IGBT junction temperature ($T_{j}$, °C) are 28.06 W, 4.42 W, 23.64 W, 150.90 A, 138.90 A, 144.90 A, and 37.38 °C, respectively.

The comparison between simulation and theoretical results shows a high degree of consistency, which confirms the validity of the developed mathematical model. The verified model is also used to generate the training dataset for the ANN that will be employed to determine the optimal variable switching frequency.

## 4. Validation of Theoretical and Simulation Results Through Experimental Data on a Designed Series DC Motor Drive Hardware

**Figure 5:**
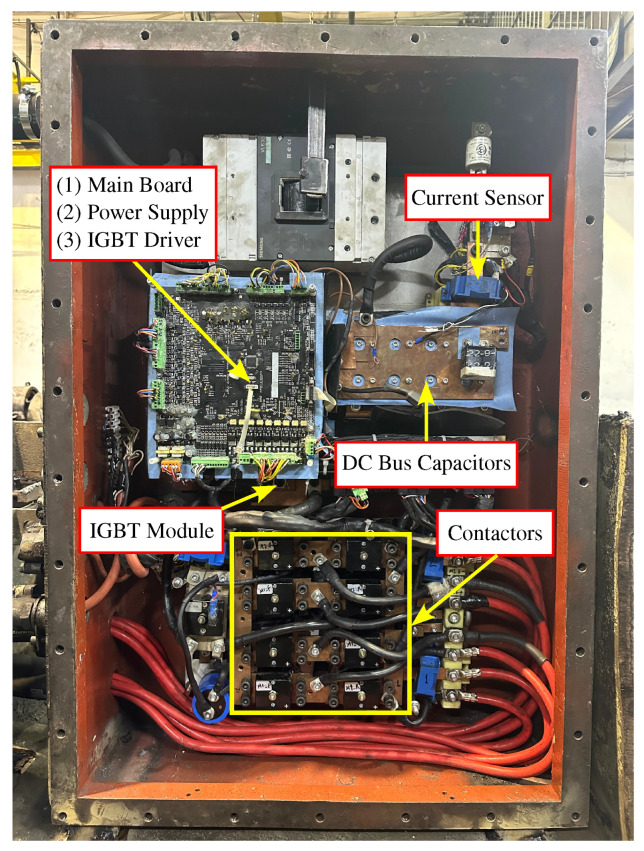
Designed motor driver hardware.

In order to validate the theoretical and simulation-based studies conducted for the series DC motor and to enable real-time performance analysis, practical field experiments were carried out using battery-powered locomotives in underground mining environments. Based on algorithms developed through theoretical and simulation efforts, a new series DC motor driver hardware was designed and manufactured as illustrated in Figure 5. The hardware architecture comprises a mainboard, power supply, IGBT driver, IGBT modules, anti-parallel diodes, a diode and capacitor group for regenerative control, a DC bus capacitor group, a pre-charge contactor group, and a direction contactor group.

The tested motor is a 21.5 kW, 96 V series-wound DC traction motor (General Electric – GE Motors, Boston, MA, USA) used in battery-powered mining locomotives. The inverter employs a 1MBI900V-120 IGBT module (Fuji Electric Co. Ltd., Tokyo, Japan), and the driver circuit uses ACPL-337 IGBT driver modules (Broadcom Inc., Irvine, CA, USA) with desaturation and Miller clamp protection. The DC-link capacitors are 250 V, 1000 µF electrolytic capacitors (TDK Electronics AG, formerly EPCOS AG, Munich, Germany) with high ripple-current endurance. Current measurement is performed using a LF 505 Hall-effect current transducer (LEM International SA, Geneva, Switzerland), while temperature feedback is provided by a 535-32AA33-103 series NTC sensor (Honeywell Sensing and Productivity Solutions, Charlotte, NC, USA). The ADC and control operations are carried out on a dsPIC33EP512MU810 microcontroller (Microchip Technology, Chandler, AZ, USA), which provides 12-bit ADC resolution and real-time PWM modulation. Voltage and temperature signals are transmitted to the microcontroller through ACPL-87B opto-isolators (Broadcom Inc., Irvine, CA, USA) after passing through operational amplifiers and low-pass filters, ensuring complete electrical isolation.

**Figure 6:**
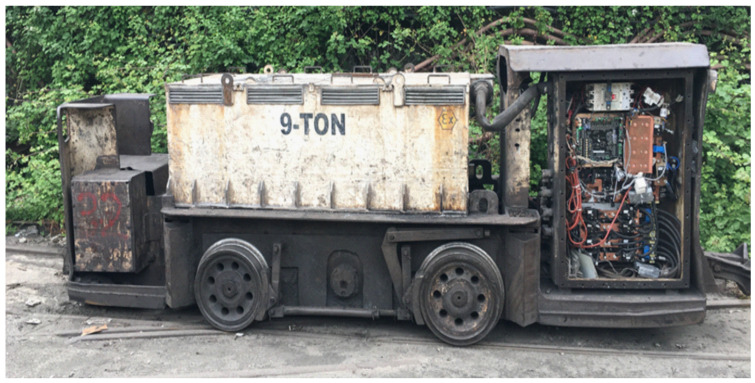
Battery-powered locomotive used for experimental validation.

To evaluate driver hardware, the field results were compared with the simulation and theoretical findings. As the first step, the rotor of the motor in the battery-powered locomotive shown in Figure 6 was locked.

**Figure 7:**
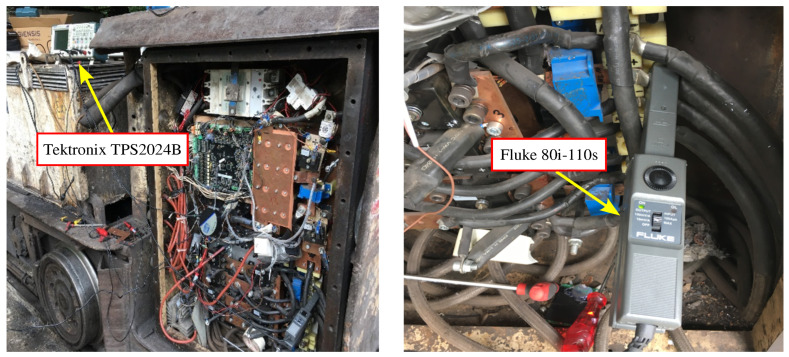
Motor current measurement setup (Tektronix TPS2024B–Fluke 80i-110s).

**Figure 8:**
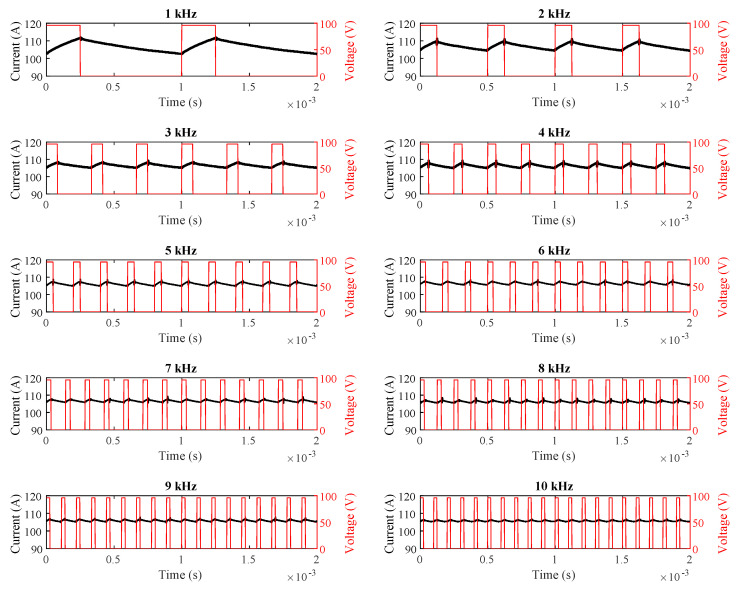
Real-time motor current waveforms at 25% duty cycle.

During these measurements, a Tektronix TPS2024B oscilloscope (Beaverton, OR, USA) and a Fluke 80i-110s current probe (Everett, WA, USA) were utilized, as shown in Figure 7. The time-domain waveforms of the motor current at switching frequencies ranging from 1 kHz to 10 kHz are given in Figure 8.

For all switching frequencies, the motor current values drawn in a constant 25% PWM duty cycle were recorded and presented in Table 3.

**Figure 9:**
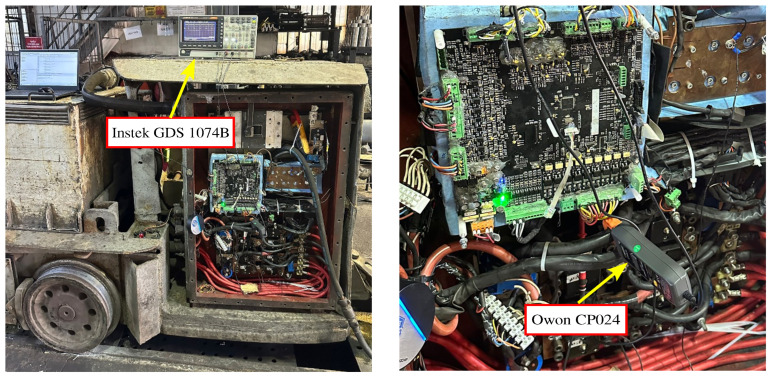
Motor current measurement setup (Instek GDS 1074B–Owon CP024).

**Figure 10:**
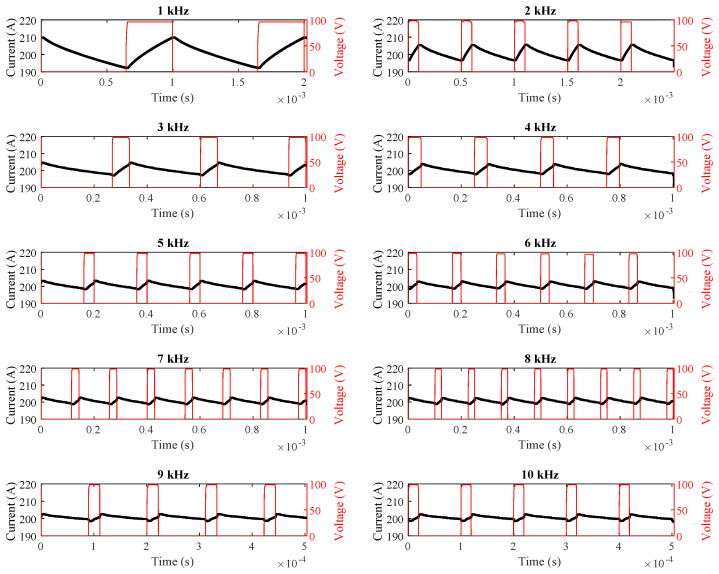
Real-time motor current waveforms for fixed 200 A current.

In the second phase of field testing, the rotor was again locked. However, instead of maintaining a constant duty cycle, the motor current was fixed at 200 A across all switching frequencies. The measured current values are presented in Table 4. Measurements were performed using an Instek GDS 1074B oscilloscope (Good Will Instrument Co., Ltd., New Taipei City, Taiwan) and an Owon CP024 current probe (Owon Technology, Xiamen, China), as illustrated in Figure 9. The corresponding current-time plots are provided in Figure 10.

As shown in Table 3 and Table 4 and Figure 8, Figure 9 and Figure 10, the simulation, field, and theoretical results exhibit close agreement under both constant duty-cycle and constant motor-current conditions across various switching frequencies.

**Figure 11:**
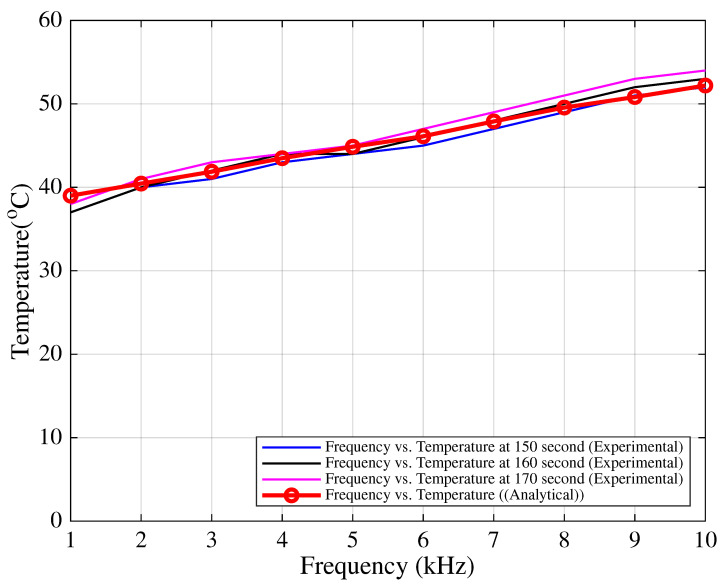
Case temperature comparison between theoretical and field measurements during 150–170 s.

Finally, the temperature measurements on the IGBT modules were performed in the field and compared with theoretical estimations. A negative temperature coefficient (NTC) sensor was placed adjacent to the IGBT case on the heatsink surface. Measurements were carried out under similar loading conditions, starting temperatures, and environmental conditions for each switching frequency in the 1–10 kHz range. The test duration was fixed at 180 s per frequency. It was observed that the measured and theoretical junction temperatures agreed notably within the 150–170 s interval, as shown in Figure 11.

## 5. Adaptive Switching Frequency Optimization via Hardware-Based Real-Time Control for Lifetime Maximization of Electronic Components in Series DC Motor Drives

This study focuses on the development of an algorithm capable of determining the optimum variable switching frequency, aiming to minimize the peak current ripple to extend the lifespan of DC link capacitors and to minimize the junction temperature oscillations to improve the IGBT’s operational life.

Temperature oscillations resulting from variable switching frequency operation are analyzed under real-world (hardware) conditions. In this context, the IGBT driver within the hardware was configured to operate in the 100 Hz–50 kHz range. During the design process, the application parameters were considered, and the limit values of the voltage slew rate (dv/dt) and the current slew rate (di/dt) were taken into account. The IGBT gate resistance ($R_{g}$) and snubber circuit components were determined based on worst-case operating conditions. Driver models supporting the Active Miller Clamp were preferred in the gate driver circuit.

The collector current of the IGBT is monitored in real time to keep the system under control. In this way, thermal and electrical stresses that may occur during frequency transitions are minimized, thus ensuring system safety.

To prevent damage to the IGBT, the switching frequency transitions are applied gradually (soft transition). The switching frequency is not changed instantaneously. In order to reduce thermal shock risk and limit abrupt electrical stresses in the circuit, the switching frequency is updated every 20 ms by increasing or decreasing it by 5% (df/dt). Furthermore, as the switching frequency changes, the sampling frequency and PWM update periods are also adjusted synchronously.

A real-time control algorithm has been developed to prevent abrupt changes in the switching frequency (F_sw_) within a PWM period. The control loop operates on a microcontroller (MC)-based system and is triggered by interrupts initiated at the midpoint of each PWM period. An interrupt (PWM_interrupt) is generated at the center of the “ON” state of each PWM period, prompting the ADC to measure system parameters such as IGBT temperature, current, and voltage. Once the ADC measurement is complete, a second interrupt (ADC_interrupt) is triggered during which the optimum target switching frequency (freqHead) for the next PWM period is computed. If the target frequency (freqHead) matches the current frequency (freqTail), no action is taken. However, if a difference exists, the freqTail is incrementally adjusted toward the freqHead within a limit defined by the maximum allowed frequency variation (freqDfDt). If the frequency gap exceeds the maximum allowed rate, freqTail is adjusted by freqDfDt; otherwise, freqHead is directly used as the new F_sw_. The updated switching frequency (F_sw_) and sampling frequency (samplingFrequency) are then written to the hardware registers for use in the next PWM cycle. The pseudocode of this algorithm is provided in Algorithm A1, which is included in Appendix A.

The selection and computation of input variables used to determine the optimum freqHead within a PWM period are critical. These inputs may include voltage, current (load), IGBT temperature, and duty cycle. Due to the complexity of formulating an objective cost function that maximizes IGBT and DC link capacitor lifetimes based on temperature and current ripple, this study employs a machine learning-based decision mechanism trained on theoretical data sets.

Before implementing the proposed machine learning-based decision mechanism to determine the optimum target switching frequency, hardware tests were conducted to ensure the dynamic and real-time update capability of F_sw_. In the initial test, IGBT temperature measurements were taken over 20 min for variable motor current values (200 A, 100 A, 150 A, 100 A, 80 A, and 50 A) under three switching frequency settings: fixed 1 kHz, fixed 10 kHz, and variable (either 1 kHz or 10 kHz). System performance at any fixed frequency between 1 kHz and 10 kHz remains within the limits defined by these two values. Therefore, the variable switching frequency were compared only with the fixed 1 kHz and 10 kHz cases. Measurements were recorded from the heat sink adjacent to the IGBT casing. Since no objective cost function could be derived at this stage, the optimum target switching frequency was determined based on minimizing the temperature fluctuation around a predefined threshold temperature value. In Algorithm A1, the function calculateTargetFrequency(inputs) was implemented using a threshold-based frequency selection method (either 1 kHz or 10 kHz), depending on the observed temperature.

**Figure 12:**
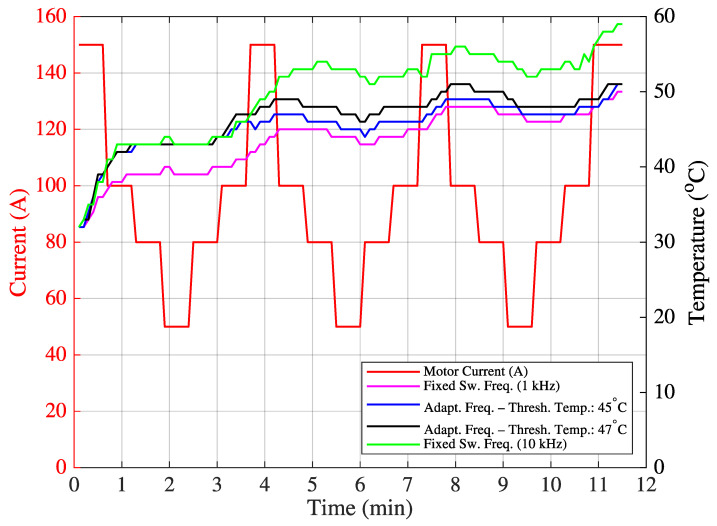
Field Test 1: IGBT temperature variation.

The threshold values were empirically set to 44 °C, 47 °C, and 51 °C. For instance, if the threshold is 44 °C, 10 kHz is selected when the temperature is below 44 °C, and 1 kHz otherwise. This threshold-based approach can be regarded as a rule-based adaptive control strategy. The resulting temperature variations are shown in Figure 12, and the corresponding statistical data (mean and standard deviation) are summarized in Table 5.

**Figure 13:**
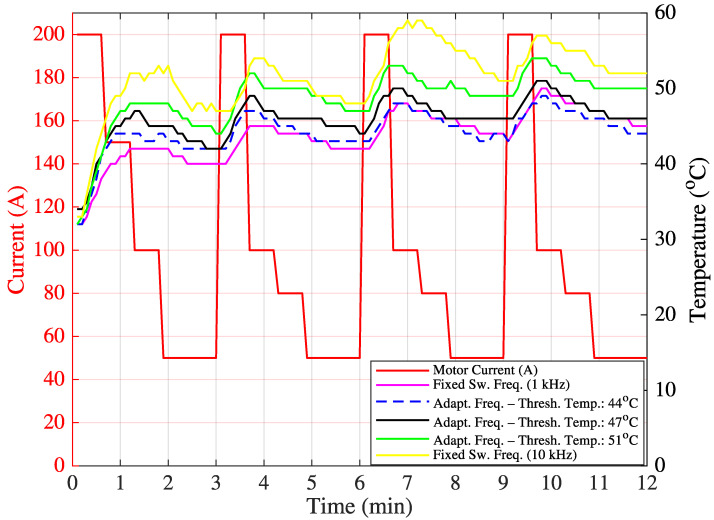
Field Test 2: IGBT temperature variation.

For a different current scenario, new threshold temperatures of 45 °C and 47 °C were defined. The corresponding temperature variations and statistics are shown in Figure 13 and Table 6, respectively.

From the results, it is observed that the use of variable switching frequency leads to a reduced standard deviation in the IGBT temperature compared to fixed switching frequencies. This implies lower thermal fluctuation and, consequently, an extended IGBT operational lifespan.

In real-world operating environments, temperature and current vary continuously, either in the same or in opposite directions. For example, one may increase while the other decreases, or both may rise or fall simultaneously. These interdependent and time-varying behaviors make the system highly complex and nonlinear, posing significant challenges for conventional threshold-based or PID-based control methods. To address these limitations, a machine learning–based frequency optimization approach using an ANN is proposed to effectively capture the nonlinear relationships among variables and determine the optimal switching frequency under dynamic operating conditions. This ANN will determine the optimum freqHead dynamically to maximize the lifetime of both the IGBT and the DC link capacitors.

## 6. ANN-Based Prediction of Optimum Switching Frequency Using Real-Time Thermal and Electrical Parameters

Based on the results obtained from field tests, it has been concluded that the instantaneous temperature variation across the IGBT must be minimized by simultaneously evaluating the instantaneous junction temperature, the PWM duty cycle, and the load (motor current). Furthermore, when motor current ripples are also considered, determining the real-time optimal switching frequency becomes a complex optimization problem. To address this, an ANN-based solution is proposed, which has been trained using a dataset derived from theoretical expressions and specified values of load, instantaneous temperature, and PWM duty cycle.

### Multi-Layer Perceptron (MLP) Network

The Multi-Layer Perceptron (MLP) is one of the most widely employed architectures in ANN, suitable for both linear and nonlinear modeling problems. An MLP consists of three main components: an input layer, one or more hidden layers, and an output layer. Each hidden layer contains *n* neurons. Computation takes place within each neuron of a given layer, and information flows forward from layer to layer via weighted interconnections. The connection weights and biases of each neuron are iteratively updated through backpropagation algorithms [49] in order to achieve the desired output.

Among various learning algorithms available in the literature, MLP is selected in this study due to its structural simplicity and efficiency in handling large-scale prediction problems. The output of a single-hidden-layer MLP can be mathematically represented as follows:

(15) $$y=f_{o}\left(\sum_{j=1}^{n}f_{h_{j}}\left(\sum_{i=1}^{m}w_{ij}x_{i}+b_{j}\right)w_{no}+b_{o}\right).$$

In (15), $x_{i}$ represents the input features and *y* is the predicted output. Here, $f_{h_{j}}$, $w_{ij}$, and $b_{j}$ correspond to the hidden layer’s activation functions, weights, and biases, respectively. Similarly, $f_{o}$, $w_{no}$, and $b_{o}$ denote the output layer’s activation function, weights, and bias. In this study, tangent sigmoid functions are employed for both the hidden and output layer activations. The training process was conducted using the MATLAB^®^ 2020a nftool module. The input features included load (motor current), instantaneous IGBT temperature, and PWM duty cycle, while the output was the optimal switching frequency. To ensure minimal temperature deviation across the IGBT (i.e., reduced thermal fluctuation), a dataset of 697 samples was generated considering maximum motor current ripples limited to 10% and PWM duty cycles varying from 10% to 90% in 10% increments. The switching frequency was swept between 1 kHz and 10 kHz during this process.

**Figure 14:**
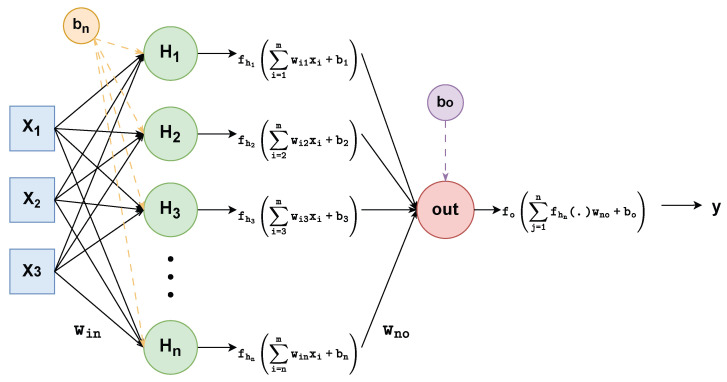
MLP Network with one hidden layer.

Figure 14 presents the detailed MLP-based ANN representation embedded within the overall system block diagram (as previously introduced in Figure 1) consisting of one input layer, one hidden layer with *n* neurons, and one output layer. In this work “tangent sigmoid” and “pure linear” functions are used as a hidden layer activation and output layer activation function. In Figure 14, $X_{1}$ corresponds to motor current (load), $X_{2}$ to PWM duty cycle, $X_{3}$ to instantaneous IGBT temperature, and the output *y* represents the optimal switching frequency.

The dataset was partitioned into 70% for training, 15% for validation, and 15% for testing. Levenberg-Marquardt and Scaled Conjugate Gradient algorithms were used as training methods. For each algorithm, three different network configurations were tested using 5, 10, and 15 neurons in the hidden layer. Training, validation, test, and overall performance metrics for all configurations are provided in Table 7.

As indicated in Table 7, the Levenberg-Marquardt algorithm with 10 neurons yielded the best test performance.

**Figure 15:**
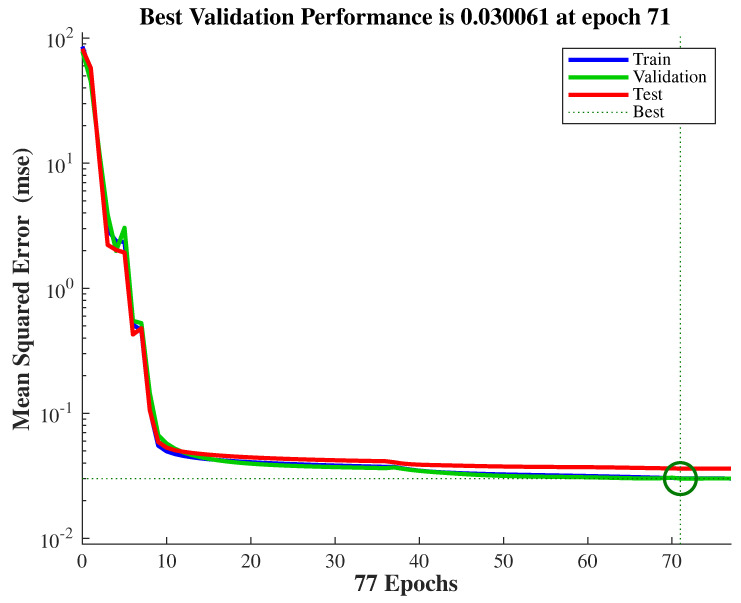
Training performance.

**Figure 16:**
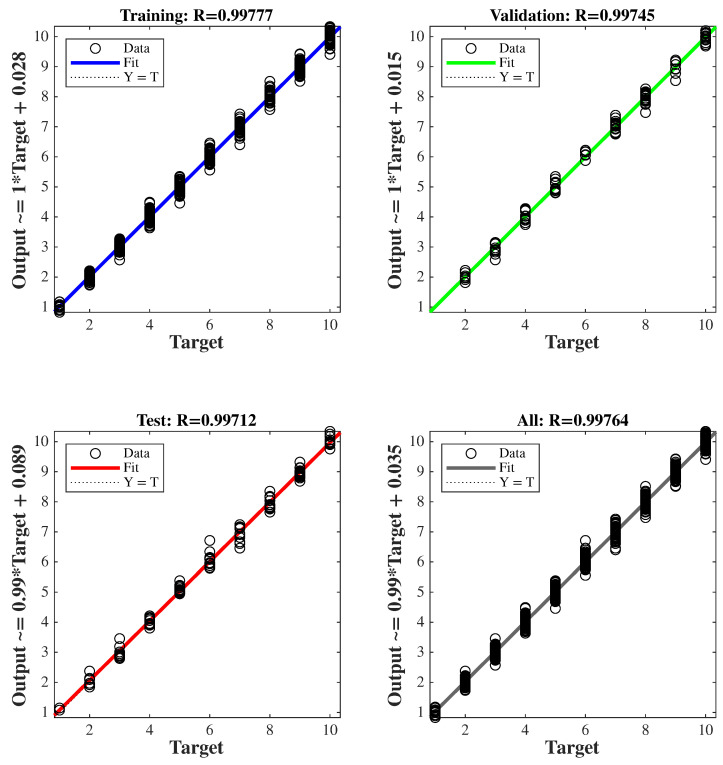
Regression plot of the network.

Although the dataset contained a limited number of samples (697), several countermeasures were implemented to prevent overfitting and ensure generalization. The dataset was randomly divided into training, validation, and testing subsets (70%, 15%, and 15%, respectively). Early stopping and dropout techniques were employed during training. In addition, the ANN architecture was deliberately kept simple with one hidden layer containing ten neurons to avoid model over-parameterization. Furthermore, the model’s predictive capability was evaluated using input combinations that were not included in the training, validation, or testing datasets, confirming its robustness across a broader input range. The training performance of the proposed ANN model is illustrated in Figure 15, showing the evolution of the mean square error (MSE) over the training epochs. To further validate the learning process, the regression analysis between the network outputs and target values is presented in Figure 16. The network achieved its optimal performance at approximately 71 epochs, yielding an MSE value of 0.003, which demonstrates the network’s high-level learning and prediction accuracy. The close agreement between the training, validation, and test performances indicates that the model exhibits no signs of overfitting, confirming the generalization capability and robustness of the proposed ANN.

In the deployed model, inputs include motor current, PWM duty cycle, and instantaneous IGBT temperature. If the predicted optimal switching frequency matches the currently applied frequency, no update is performed. Otherwise, the system updates the switching frequency to the newly predicted optimal value. Within the hardware, this replacement is implemented by substituting the calculateTargetFrequency(inputs) function in Algorithm A1 with the ANN output. The corresponding pseudocode, referred to as Algorithm A2, is given in Appendix B.

The embedded code developed for the hardware implementation of the ANN algorithm utilizes approximately 3 KB of ROM and 200 bytes of RAM. The implementation is based on the dsPIC33EP512MU810 digital signal controller and utilizes the Q16.15 fixed-point format, along with built-in DSP functions, to achieve computational efficiency. Through a statically allocated memory architecture, the optimized implementation enables the ANN algorithm to execute in approximately 30 microseconds at 70 MIPS. As a result of this level of optimization, the function can operate continuously even at the maximum switching frequency (10 kHz) without encountering any hardware or timing limitations. Consequently, the developed embedded ANN module is highly optimized in terms of both computational performance and memory utilization, providing an effective solution for real-time control applications.

## 7. Comparative Analysis of Thermal and Lifetime Performance of ANN-Controlled and Fixed Switching Frequency Strategies Under Varying Load and Duty Cycle Conditions

The function mlp_model.predict corresponds to the analytical expression provided in Equation (15), as defined in Algorithm A2. On the hardware side, the optimal switching frequency is computed in real time by substituting the input-hidden layer weights, hidden layer biases, and input variables obtained from the training phase into Equation (15).

**Figure 17:**
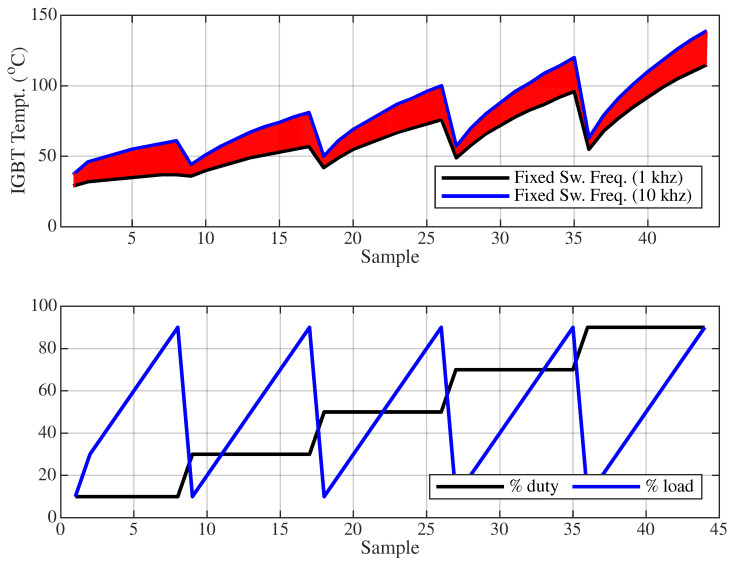
IGBT temperature variation under different switching frequencies and load/duty cycle conditions.

Figure 17 illustrates the variation of IGBT temperature at 1 kHz and 10 kHz switching frequencies under test conditions with the trained ANN, using a different dataset in which the load and duty cycle values are increased by 10% and 20%, respectively. In this graph, the black curve represents the 1 kHz switching case, while the blue curve corresponds to the 10 kHz case. The red-shaded area between these curves indicates the temperature range that can be regulated by a variable switching frequency based on the corresponding load and duty cycle levels.

Based on the red-shaded region in Figure 17, two different scenarios are defined, where the IGBT temperature ranges are 32–77 °C and 58–140 °C, respectively. In both scenarios, 500 sample points are randomly generated based on arbitrary load and duty cycle combinations. Due to randomness, consecutive samples within a scenario may cause instantaneous two-way changes between the minimum and maximum temperature values. For instance, in Scenario 1, the IGBT temperature can suddenly vary from 32 °C to 77 °C or vice versa. Although such abrupt changes are unlikely to occur under practical operating conditions, randomly generated samples were intentionally used to stress-test the ANN-based variable switching frequency approach.

For each scenario, the ANN-determined optimal (adaptive) switching frequency is compared against fixed switching frequencies between 1 kHz and 10 kHz in terms of three criteria: Average Capacitor Lifetime (Years), Average IGBT Lifetime (Cycles), and Total IGBT Power Loss (W).

**Figure 18:**
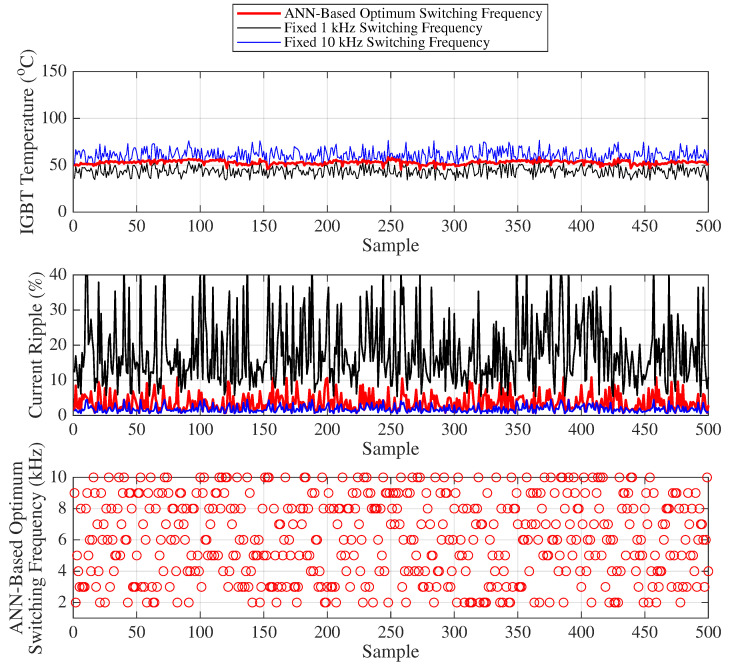
Scenario 1: IGBT temperature, motor current ripple, and optimum frequency.

Figure 18 presents the variation of the IGBT temperature, motor current ripple, and the optimal frequency predicted by the ANN for 500 randomly selected samples, where load and duty cycle values are chosen such that the IGBT temperature range varies between 32 °C and 77 °C for Scenario 1. To avoid visual complexity, only two fixed switching frequencies (1 kHz and 10 kHz) are compared with the ANN-based optimum switching frequency approach in Figure 18 (statistical results for other frequencies are provided in the table).

In the first graph of Figure 18, IGBT temperature (°C) variations are shown for different switching frequency strategies under randomly selected load and duty cycle conditions. For fixed 1 kHz operation (black line), the IGBT temperature varies between 32.16 °C and 57.52 °C, whereas for 10 kHz (blue line), it changes between 45.26 °C and 76.65 °C (For other frequencies, temperature values are expected to remain within this range). When using the ANN-based optimum switching frequency (red line), the temperature changes between 44.25 °C and 59.60 °C. The difference between the minimum and maximum temperature (temperature fluctuation) is 15.35 °C for the ANN-based method, while it is 25.35 °C and 31.39 °C for the fixed 1 kHz and 10 kHz operations, respectively. This temperature fluctuation is a major factor influencing IGBT lifetime. The graph clearly shows that the temperature variation under the ANN-based optimum switching frequency (red line) exhibits significantly smaller fluctuation.

Similarly, the middle graph of Figure 18 illustrates the current ripple (%) variations for different switching frequency strategies under randomly selected load and duty cycle conditions. For the fixed 1 kHz operation (black line), the current ripple varies between 5.41% and 45.08%, while for 10 kHz (blue line) it changes between 0.54% and 4.51%. (For other frequencies, the ripple values are expected to remain within this range.) With the ANN-based optimum switching frequency (red line), the ripple varies between 0.66% and 10.82%. The difference between the minimum and maximum ripple is 10.16%, compared to 39.67% and 3.97% for the fixed 1 kHz and 10 kHz operations, respectively. Since the study aims to limit the maximum motor current ripple to 10%, it is evident from the graph that the ANN-based optimum switching frequency (red circles) maintains the current ripple within this desired range.

Finally, the lower graph of Figure 18 presents the ANN-based optimum switching frequencies corresponding to the 500 randomly selected samples, based on the respective load, duty cycle, and temperature inputs.

Table 8 and Table 9 provide the mean, standard deviation, minimum–maximum values, and their respective differences for both current ripple and IGBT temperature under variable and fixed switching frequencies (such as between 1 kHz and 10 kHz). These statistical metrics are used to derive the capacitor lifetime (years), IGBT lifetime (cycles), and average IGBT power losses (watts) given Table 10.

To ensure a fair comparison of the effects of variable and fixed switching frequencies on the lifetimes of the IGBT and the capacitor, a fairness index (ζ) is defined as in (16). The fairness index for any given frequency is defined in (17) and (18) as the ratio of the IGBT or capacitor lifetime at that frequency to the maximum lifetime observed among all frequencies. The fairness index value varies from 0 to 1. Thus, as the fairness increases, the fairness index grows higher.

(16) $$\zeta_{\mathrm{total}}^{\left(i\right)}=\zeta_{\mathrm{IGBT}}^{\left(i\right)}\times \zeta_{\mathrm{Cap}}^{\left(i\right)}\;$$

where *i* indicates variable and fixed switching frequencies (between 1 to 10 kHz). $\zeta_{\mathrm{IGBT}}^{\left(i\right)}$ and $\zeta_{\mathrm{Cap}}^{\left(i\right)}$ are defined as the fairness indices of IGBT and capacitor lifetimes, respectively.

(17) $$\zeta_{\mathrm{IGBT}}^{\left(i\right)}=\frac{\mathrm{Avg}.\;{\mathrm{IGBT}\_\mathrm{Lifetime}}^{\left(i\right)}}{\max_{i}\left(\mathrm{Avg}.\;{\mathrm{IGBT}\_\mathrm{Lifetime}}^{\left(i\right)}\right)}$$

(18) $$\zeta_{\mathrm{Cap}}^{\left(i\right)}=\frac{\mathrm{Avg}.\;{\mathrm{Cap}\_\mathrm{Lifetime}}^{\left(i\right)}}{\max_{i}\left(\mathrm{Avg}.\;{\mathrm{Cap}\_\mathrm{Lifetime}}^{\left(i\right)}\right)}$$

Fairness indices for variable and fixed switching frequencies are given in the Table 11.

In terms of the fairness index, the use of variable switching frequency exhibits approximately 13 times higher performance compared to the closest fixed switching frequency, which is 2 kHz.

**Figure 19:**
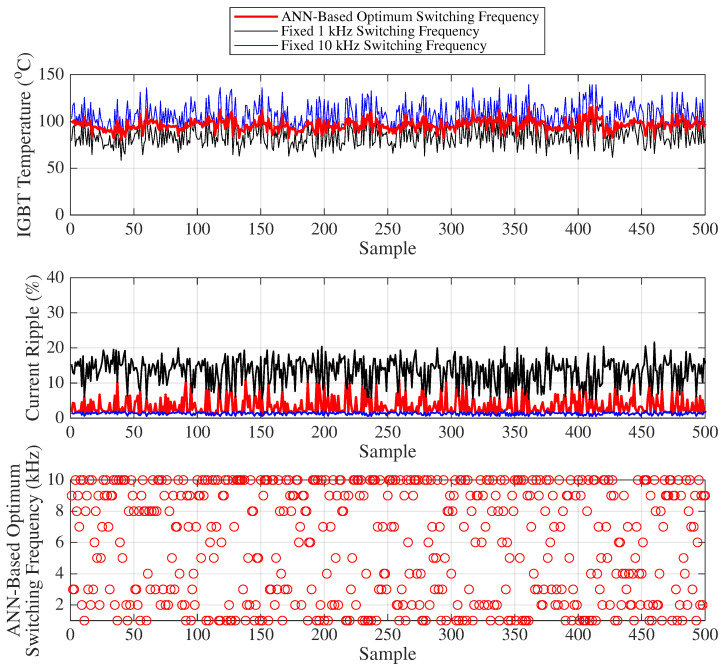
Scenario 2: IGBT temperature, motor current ripple, and optimum frequency.

Figure 19 presents the variation of the IGBT temperature, motor current ripple, and the optimal frequency predicted by the ANN for 500 randomly selected samples, where the load and duty cycle values are chosen such that the IGBT temperature range varies between 58 °C and 140 °C for Scenario 2, representing higher IGBT temperature conditions compared to Scenario 1. The analyses and interpretations for the graphs in Figure 19 can be carried out in a similar manner to those described for Figure 18.

Table 12 and Table 13 provide the mean, standard deviation, minimum–maximum values, and their respective differences for both current ripple and IGBT temperature under variable and fixed switching frequencies (such as between 1 kHz and 10 kHz). These statistical metrics are used to derive the capacitor lifetime (years), IGBT lifetime (cycles), and average IGBT power losses (watts) given Table 14. Fairness indices for variable and fixed switching frequencies are given in the Table 15.

In terms of the fairness index, the use of variable switching frequency exhibits approximately 10 times higher performance compared to the closest fixed switching frequency, which is 1 kHz.

The analysis of the above results reveals that an increase in either the IGBT temperature or its fluctuation significantly reduces the average lifetime of the IGBT. It has been observed that employing a variable switching frequency effectively lowers both the average IGBT temperature and its thermal fluctuations compared to the fixed switching frequency operation, thereby extending the average IGBT lifetime. Similarly, when a variable switching frequency is applied, the current ripple in the motor converges towards the levels seen in fixed 10 kHz operation. As a result, the average capacitor lifetime is improved compared to the use of a fixed low switching frequency.

**Figure 20:**
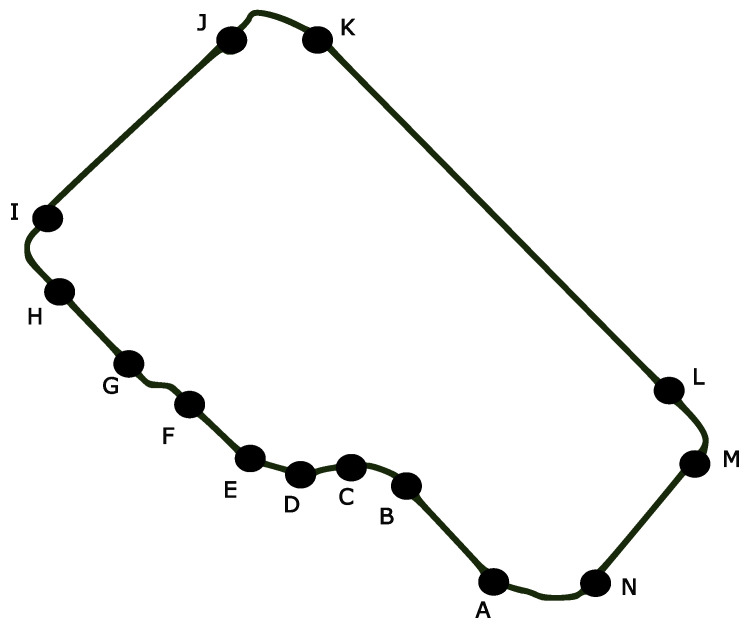
Real-world operation route defined for the battery-powered locomotive [50].

In addition to the evaluations conducted using randomly generated load and duty cycle values, a realistic operating route was defined to simulate the actual conditions under which the electric locomotive might operate. Based on the route shown in Figure 20, parameters such as locomotive load, road slope, segment lengths, turning angles, speeds, and times required to reach nominal velocity were specified [50]. The equations used to compute the required motor power, motor load, motor current, and PWM duty cycle during locomotive motion are provided in Appendix C.

In the field test scenarios, the operating characteristics of the locomotive under both full-load and half-load conditions are presented in detail. Under full-load operation, which corresponds to fully loaded wagons, the locomotive was assumed to reach a maximum speed of 10 km/h. Under half-load conditions, the locomotive speed was assumed to increase by approximately 20% relative to the full-load case. This limit reflects practical railway safety considerations, as speed increments beyond 20% may increase the risk of derailment; therefore, the speed variation was constrained to remain within realistic and safe operational boundaries. Furthermore, even if the locomotive speed were to remain unchanged at half load or vary within these practical limits, the proposed approach consistently exhibits similar trends in optimal switching frequency and overall system performance. As a result, such variations do not affect the analysis or the conclusions drawn from the study. Accordingly, the analysis in this study focuses on the half-load and full-load operating conditions, which effectively represent the system behavior relevant to the research objectives.

As the first scenario, the fully loaded wagon condition is considered, and Table 16 presents the relevant parameters, including the required power, calculated motor load, and the corresponding PWM duty cycle values.

**Figure 21:**
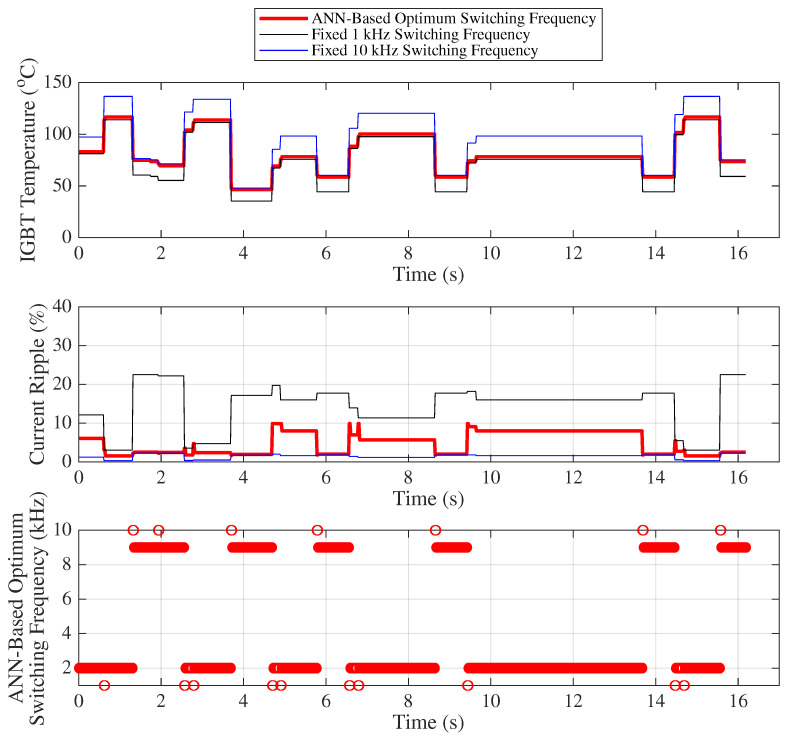
Full-load condition: variation of IGBT temperature, motor current ripple, and ANN-predicted optimal switching frequency.

In Figure 21, the variations in IGBT temperature, motor current ripple, and the optimal switching frequency obtained by ANN are presented under full load conditions. Table 17 and Table 18 provide the mean, standard deviation, minimum–maximum values, and their respective differences for both current ripple and IGBT temperature under variable and fixed frequency values. These statistical metrics are used to derive the capacitor lifetime (years), IGBT lifetime (cycles), and average IGBT power losses (watts) given Table 19. Fairness indices for variable and fixed switching frequencies are given in the Table 20.

In the second field scenario, the load carried by the locomotive (representing half-filled wagons) was reduced by half. For this scenario, certain parameters, the required power, the corresponding calculated motor load and PWM duty cycle values are presented in Table 21.

**Figure 22:**
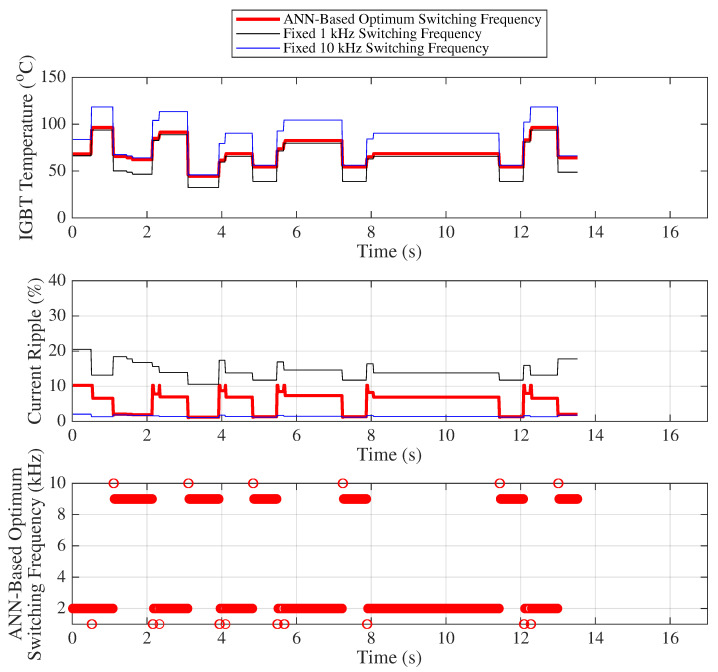
Half-load condition: variation of IGBT temperature, motor current ripple, and ANN-predicted optimal switching frequency.

In Figure 22, the variations in IGBT temperature, motor current ripple, and the optimal switching frequency obtained by ANN are presented under half load conditions. Table 22 and Table 23 provide the mean, standard deviation, minimum–maximum values, and their respective differences for both current ripple and IGBT temperature under variable and fixed frequency values. These statistical metrics are used to derive the capacitor lifetime (years), IGBT lifetime (cycles), and average IGBT power losses (watts) given Table 24. Fairness indices for variable and fixed switching frequencies are given in the Table 25.

In terms of the fairness index, the use of variable switching frequency exhibits approximately 2 times higher performance compared to the closest fixed switching frequency, which is 2 kHz according to the results obtained from both Table 20 and Table 25.

## 8. Conclusions

This study has presented an ANN-based adaptive switching frequency control strategy for series DC motor drives in battery-powered mining locomotives, designed to extend the lifetime and improve the reliability of key power-electronic components such as IGBTs and DC bus capacitors. Within this framework, the primary challenges associated with conventional fixed switching frequencies—namely, excessive motor current ripple at low frequencies and increased IGBT thermal stress at high frequencies—were effectively addressed through a machine learning–based adaptive control approach. The proposed algorithm was trained using data acquired from current sensors, NTC temperature sensors, and a potentiometer defining the target current (PWM duty). By enabling real-time decision making, the ANN model predicts the optimal switching frequency that minimizes both IGBT thermal fluctuations and motor current ripple. The experimental and simulation results demonstrate that the adaptive algorithm maintains an average current ripple of less than 5% and a maximum of 10%, while significantly reducing temperature variations across the IGBT module. A fairness index was employed to quantitatively assess the lifetime improvements achieved under variable switching frequency operation. According to this index, the proposed adaptive control strategy enhances the overall system reliability and component lifetime by up to 13 times compared with fixed-frequency operation. In addition, the proposed adaptive control approach contributes to improved energy efficiency and sustainability by reducing IGBT switching losses and overall power dissipation. These results confirm that the integration of embedded machine learning, sensor-based signal processing, and adaptive control algorithms can substantially improve the efficiency and durability of power-electronic systems in real-time industrial applications. Moreover, this integration holds great potential for broader real-time applications, including autonomous vehicles, sensor fusion, monitoring, state estimation, and fault diagnosis, where adaptive and data-driven control play a critical role. Future work will focus on implementing this approach within industrial drive controllers, extending it to multi-motor configurations, and exploring online learning capabilities for adaptive frequency tuning under dynamic operating conditions.

## Figures and Tables

**Table 1:** Electrical specifications of the designed system.

Specification	Value
Battery nominal voltage	96 V
Nominal DC current	200 A
Maximum switching frequency	10 kHz

**Table 2:** Specifications of the series DC motor.

Parameter	Value
Rated power	21.5 kW
Rated voltage	119 VDC
Armature current ($I_{a}$)	223 A
Field current ($I_{f}$)	223 A
Rated speed	1022 r/min
Armature resistance ($R_{a}$)	0.023 Ω
Armature inductance ($L_{a}$)	0.000745 H
Field resistance ($R_{f}$)	0.06 Ω
Field inductance ($L_{f}$)	0.000280 H

**Table 3:** Motor current values in amperes (A) at 25% duty cycle: Simulation, Experimental, and Theoretical.

Frequency	Simulation		Field		Theory	
**(kHz)**	$I_{\min}$	$I_{\max}$	$I_{\min}$	$I_{\max}$	$I_{\min}$	$I_{\max}$
1	98.2	116.5	102.5	111.9	95.9	117.7
2	102.8	111.8	104.4	109.8	101.3	112.2
3	104.4	110.4	105.0	109.2	103.1	110.4
4	105.1	109.6	105.0	109.0	104.0	109.5
5	105.5	109.1	105.0	108.6	104.6	108.9
6	105.9	108.7	105.4	108.4	104.9	108.6
7	106.1	108.5	105.6	108.2	105.2	108.3
8	106.2	108.4	105.5	107.3	105.4	108.1
9	106.4	108.3	105.2	106.8	105.5	108.0
10	106.4	108.2	105.2	106.6	105.7	107.8

**Table 4:** Motor current values in amperes (A) for 200 A condition: Simulation, Field, and Theory.

Frequency	Simulation		Field		Theory	
**(kHz)**	$I_{\min}$	$I_{\max}$	$I_{\min}$	$I_{\max}$	$I_{\min}$	$I_{\max}$
1	191.18	208.82	192.53	209.24	189.12	210.88
2	195.18	204.82	196.84	204.97	194.56	205.44
3	196.33	203.67	197.60	204.17	196.37	203.63
4	197.44	202.56	198.23	203.29	197.28	202.72
5	197.46	202.54	198.68	202.73	197.82	202.18
6	197.99	202.01	198.95	202.53	198.19	201.81
7	198.13	201.87	199.26	202.25	198.45	201.55
8	198.29	201.71	199.48	202.04	198.64	201.36
9	198.35	201.65	198.92	202.30	198.79	201.21
10	198.12	201.88	198.77	202.15	198.91	201.09

**Table 5:** Field Test 1: Statistical data of IGBT temperature variation.

Threshold	Fixed Frequency		Variable Frequency		
	**1 kHz**	**10 kHz**	**44 °C**	**47 °C**	**51 °C**
Mean (°C)	43.76	51.50	44.28	45.88	48.66
Std Dev (°C)	3.41	4.53	2.80	2.91	3.78

**Table 6:** Field Test 2: Statistical data of IGBT temperature variation.

Threshold	Fixed Frequency		Variable Frequency	
	**1 kHz**	**10 kHz**	**44 °C**	**47 °C**
Mean (°C)	43.49	49.85	45.44	46.55
Std Dev (°C)	4.02	5.92	3.33	3.79

**Table 7:** Performance comparison of MLP configurations.

Algorithm	# Neurons	Training	Validation	Testing	Overall
Levenberg–Marquardt	5	0.9827	0.9815	0.9750	0.9815
Scaled Conjugate Gradient	5	0.9633	0.9906	0.9579	0.9645
**Levenberg–Marquardt**	**10**	**0.9977**	**0.9974**	**0.9971**	**0.9976**
Scaled Conjugate Gradient	10	0.9803	0.9826	0.9618	0.9782
Levenberg–Marquardt	15	0.9727	0.9797	0.9785	0.9745
Scaled Conjugate Gradient	15	0.9630	0.9885	0.9807	0.9680

**Table 8:** Scenario 1: Statistics of motor current ripple values.

Frequency (kHz)	Var. Freq.	1	2	3	4	5	6	7	8	9	10
Mean	3.73	18.80	9.40	6.27	4.70	3.76	3.13	2.69	2.35	2.09	1.88
Std. Dev.	2.41	10.16	5.08	3.38	2.54	2.03	1.69	1.45	1.27	1.13	1.02
Min	0.66	5.41	2.71	1.80	1.35	1.08	0.90	0.77	0.68	0.60	0.54
Max	10.82	45.08	22.54	15.03	11.27	9.02	7.51	6.44	5.64	5.01	4.51
ΔA	10.16	39.67	19.84	13.23	9.92	7.94	6.61	5.67	4.96	4.41	3.97

**Table 9:** Scenario 1: Statistics of IGBT temperature values.

Frequency (kHz)	Var. Freq.	1	2	3	4	5	6	7	8	9	10
Mean	52.86	44.07	45.89	47.72	49.54	51.37	53.19	55.02	56.84	58.67	60.49
Std. Dev.	1.90	4.97	4.96	5.02	5.12	5.28	5.48	5.73	6.01	6.32	6.67
Min	44.25	32.16	33.70	35.25	36.79	38.33	39.87	41.42	42.96	44.37	45.26
Max	59.60	57.52	59.60	61.69	63.78	65.86	67.95	70.04	72.13	74.38	76.65
ΔT	15.35	25.35	25.90	26.44	26.99	27.53	28.08	28.63	29.17	30.01	31.39

**Table 10:** Scenario 1: Average capacitor and IGBT lifetime, average IGBT losses.

Frequency (kHz)	Var. Freq.	1	2	3	4	5	6	7	8	9	10
Avg. Capacitor Life (years)	10.91	5.19	8.33	9.76	10.56	11.07	11.42	11.68	11.88	12.04	12.17
Avg. IGBT Life (cycles) ($\times 10^{9}$)	19,857	2505	1927	1486	1151	895	698	547	430	321	219
Avg. IGBT Losses (W)	60.39	41.35	45.29	49.25	53.20	57.16	61.12	65.07	69.03	72.98	76.94

**Table 11:** Scenario 1: Fairness index.

Frequency (kHz)	Var. Freq.	1	2	3	4	5	6	7	8	9	10
Fairness index $\left(\zeta_{\mathrm{total}}\right)$	0.896	0.054	0.066	0.060	0.050	0.041	0.033	0.026	0.021	0.016	0.011

**Table 12:** Scenario 2: Statistics of motor current ripple values.

Frequency (kHz)	Var. Freq.	1	2	3	4	5	6	7	8	9	10
Mean	3.35	13.29	6.64	4.43	3.32	2.66	2.21	1.90	1.66	1.48	1.33
Std. Dev.	2.35	3.76	1.88	1.25	0.94	0.75	0.63	0.54	0.47	0.42	0.38
Min	0.81	5.41	2.71	1.80	1.35	1.08	0.90	0.77	0.68	0.60	0.54
Max	10.91	21.94	10.97	7.31	5.48	4.39	3.66	3.13	2.74	2.44	2.19
ΔA	10.10	16.53	8.26	5.51	4.13	3.30	2.75	2.36	2.07	1.84	1.65

**Table 13:** Scenario 2: Statistics of IGBT temperature values.

Frequency (kHz)	Var. Freq.	1	2	3	4	5	6	7	8	9	10
Mean	96.07	85.14	87.41	89.68	91.94	94.21	96.47	98.73	100.99	103.26	105.52
Std. Dev.	6.44	12.37	12.51	12.64	12.78	12.93	13.08	13.24	13.40	13.57	13.75
Min	80.11	58.53	60.96	63.40	65.84	68.28	70.72	73.16	75.60	78.02	80.11
Max	115.20	115.20	117.89	120.57	123.24	125.91	128.59	131.26	133.93	136.60	139.28
ΔT	35.08	56.67	56.93	57.16	57.40	57.63	57.87	58.10	58.33	58.58	59.16

**Table 14:** Scenario 2: Average capacitor and IGBT lifetime, average IGBT losses.

Frequency (kHz)	Var. Freq.	1	2	3	4	5	6	7	8	9	10
Avg. Capacitor Life (years)	10.37	5.13	8.28	9.72	10.53	11.04	11.40	11.66	11.87	12.03	12.15
Avg. IGBT Life (cycles) ($\times 10^{9}$)	11.40	1.60	1.35	1.15	0.97	0.83	0.71	0.61	0.52	0.45	0.37
Avg. IGBT Losses (W)	154.06	130.36	135.30	140.21	145.12	150.02	154.93	159.83	164.74	169.65	174.55

**Table 15:** Scenario 2: Fairness index.

Frequency (kHz)	Var. Freq.	1	2	3	4	5	6	7	8	9	10
Fairness index $\left(\zeta_{\mathrm{total}}\right)$	0.853	0.059	0.081	0.080	0.074	0.066	0.058	0.051	0.045	0.039	0.033

**Table 16:** Parameter variations based on the route profile under full-load wagon conditions.

Route	Length	Slope	Init. Speed	Final Speed	Avg. Time	Req. Power	Motor Load	Duty Cycle
	**(m)**	**(°)**	**(km/h)**	**(km/h)**	**(s)**	**(W)**	**(%)**	**(%)**
A-B	56	1.64	0	10	40.32	16,741	84	40
A-B	118	1.64	10	10	42.48	20,088	100	79
B-C	36	1.74	5	5	25.92	10,396	52	40
C-D	15	1.65	5	5	10.80	10,011	50	40
D-E	53	1.39	5	5	38.16	8898	44	40
E-F	28	1.19	5	10	13.44	19,062	95	59
E-F	154	1.19	10	10	55.44	18,376	92	79
F-G	50	0.90	3	3	60.00	3508	18	24
G-H	22.4	0.0024	3	10	12.41	11,097	55	51
G-H	147.6	0.0024	10	10	53.14	10,692	53	79
H-I	65	0.56	5	5	46.80	5346	27	40
I-J	28	0.56	5	10	13.44	15,017	75	59
I-J	312	0.56	10	10	112.32	15,465	77	79
J-K	65	0.56	5	5	46.80	5346	27	40
K-L	28	0.0024	5	10	13.44	11,437	57	59
K-L	672	0.0024	10	10	241.92	10,692	53	79
L-M	65	0.56	5	5	46.80	5346	27	40
M-N	28	1.65	5	10	13.44	18,484	92	59
M-N	146	1.65	10	10	52.56	20,088	100	79
N-A	51	1.65	5	5	36.72	10,011	50	40

**Table 17:** Full-load condition: Statistics of motor current ripple values.

Frequency (kHz)	Var. Freq.	1	2	3	4	5	6	7	8	9	10
Mean	4.67	14.21	7.10	4.74	3.55	2.84	2.37	2.03	1.78	1.58	1.42
Std. Dev.	2.76	5.74	2.87	1.91	1.43	1.15	0.96	0.82	0.72	0.64	0.57
Min	1.52	3.05	1.52	1.02	0.76	0.61	0.51	0.44	0.38	0.34	0.30
Max	9.88	22.54	11.27	7.51	5.64	4.51	3.76	3.22	2.82	2.50	2.25
ΔA	8.36	19.49	9.75	6.50	4.87	3.90	3.25	2.79	2.44	2.17	1.95

**Table 18:** Full-load condition: Statistics of IGBT temperature values.

Frequency (kHz)	Var. Freq.	1	2	3	4	5	6	7	8	9	10
Mean	81.88	75.79	77.98	80.18	82.37	84.56	86.76	88.95	91.14	93.34	95.53
Std. Dev.	20.03	23.99	24.31	24.61	24.92	25.24	25.55	25.86	26.18	26.50	26.82
Min	46.46	35.43	36.80	38.18	39.56	40.94	42.32	43.70	45.08	46.46	47.84
Max	116.59	114.07	116.59	119.09	121.60	124.10	126.61	129.11	131.61	134.12	136.62
ΔT	70.12	78.64	79.78	80.91	82.03	83.16	84.28	85.41	86.53	87.65	88.78

**Table 19:** Full-load condition: Average capacitor and IGBT lifetime, average IGBT losses.

Frequency (kHz)	Var. Freq.	1	2	3	4	5	6	7	8	9	10
Avg. Capacitor Life (years)	9.45	5.11	8.27	9.71	10.52	11.03	11.39	11.66	11.86	12.02	12.15
Avg. IGBT Life (cycles) ($\times 10^{9}$)	609.00	483.31	385.93	309.47	248.91	200.76	162.38	131.68	107.06	87.27	71.32
Avg. IGBT Losses (W)	123.29	110.09	114.86	119.61	124.37	129.12	133.88	138.63	143.38	148.14	152.89

**Table 20:** Full-load condition: Fairness index.

Frequency (kHz)	Var. Freq.	1	2	3	4	5	6	7	8	9	10
Fairness index $\left(\zeta_{\mathrm{total}}\right)$	0.778	0.334	0.431	0.406	0.354	0.299	0.250	0.207	0.172	0.142	0.117

**Table 21:** Parameter variations based on the route profile under half-load wagon conditions.

Route	Length	Slope	Init. Speed	Final Speed	Avg. Time	Req. Power	Motor Load	Duty Cycle
	**(m)**	**(°)**	**(km/h)**	**(km/h)**	**(s)**	**(W)**	**(%)**	**(%)**
A-B	56	1.64	0	12	33.60	11,165	56	47
A-B	118	1.64	12	12	35.40	13,203	95	95
B-C	36	1.74	6	6	21.60	6524	33	47
C-D	15	1.65	6	6	9.00	6293	31	47
D-E	53	1.39	6	6	31.80	5626	28	47
E-F	28	1.19	6	12	11.20	12,708	64	71
E-F	154	1.19	12	12	46.20	12,176	61	95
F-G	50	0.90	3.6	3.6	50.00	2208	11	28
G-H	22.4	0.0024	3.6	12	10.34	7902	40	62
G-H	147.6	0.0024	12	12	44.28	7566	38	95
H-I	65	0.56	6	6	39.00	3495	17	47
I-J	28	0.56	6	12	11.20	10,282	51	71
I-J	312	0.56	12	12	93.60	10,430	52	95
J-K	65	0.56	6	6	39.00	3495	17	47
K-L	28	0.0024	6	12	11.20	8134	41	71
K-L	672	0.0024	12	12	201.60	7566	38	95
L-M	65	0.56	6	6	39.00	3495	17	47
M-N	28	1.65	6	12	11.20	12,362	62	71
M-N	146	1.65	12	12	43.80	13,203	95	95
N-A	51	1.65	6	6	30.60	6293	31	47

**Table 22:** Half-load condition: Statistics of motor current ripple values.

Frequency (kHz)	Var. Freq.	1	2	3	4	5	6	7	8	9	10
Mean	5.35	14.19	7.10	4.73	3.55	2.84	2.37	2.03	1.77	1.58	1.42
Std. Dev.	2.79	2.29	1.14	0.76	0.57	0.46	0.38	0.33	0.29	0.25	0.23
Min	1.06	10.55	5.28	3.52	2.64	2.11	1.76	1.51	1.32	1.17	1.06
Max	10.25	20.50	10.25	6.83	5.12	4.10	3.42	2.93	2.56	2.28	2.05
ΔA	9.19	9.95	4.97	3.32	2.49	1.99	1.66	1.42	1.24	1.11	0.99

**Table 23:** Half-load condition: Statistics of IGBT temperature values.

Frequency (kHz)	Var. Freq.	1	2	3	4	5	6	7	8	9	10
Mean	70.21	63.52	65.92	68.31	70.71	73.10	75.49	77.89	80.28	82.68	85.07
Std. Dev.	14.08	18.45	18.82	19.18	19.55	19.92	20.29	20.67	21.05	21.42	21.81
Min	44.38	32.46	33.95	35.44	36.93	38.42	39.91	41.40	42.89	44.38	45.88
Max	96.45	93.69	96.45	99.20	101.94	104.69	107.44	110.18	112.93	115.67	118.42
ΔT	52.07	61.23	62.50	63.76	65.01	66.27	67.52	68.78	70.03	71.29	72.55

**Table 24:** Half-load condition: Average capacitor and IGBT lifetime, average IGBT losses.

Frequency (kHz)	Var. Freq.	1	2	3	4	5	6	7	8	9	10
Avg. Capacitor Life (years)	8.61	4.49	7.75	9.30	10.18	10.76	11.15	11.45	11.67	11.85	11.99
Avg. IGBT Life (cycles) ($\times 10^{9}$)	6817.2	4470.7	3367.3	2551.0	1941.6	1484.2	1139.4	878.3	679.8	528.1	411.9
Avg. IGBT Losses (W)	98.00	83.51	88.70	93.89	99.08	104.27	109.46	114.65	119.84	125.03	130.23

**Table 25:** Half-load condition: Fairness index.

Frequency (kHz)	Var. Freq.	1	2	3	4	5	6	7	8	9	10
Fairness index $\left(\zeta_{\mathrm{total}}\right)$	0.718	0.246	0.319	0.290	0.242	0.195	0.155	0.123	0.097	0.077	0.060

## Data Availability

The original contributions presented in this study are included in the article. Further inquiries can be directed to the corresponding authors.

## Appendix A. Variable Switching Frequency Usage

**Algorithm A1** Variable Switching Frequency Usage
1: **function** onPwmInterrupt 2: // (1) At the midpoint of the on duration of each PWM period, the microcontroller generates an interrupt (PWM_interrupt). 3: // When the PWM interrupt is triggered, activate the ADC unit. 4: triggerAdc() 5: **end function** ** ** 6: **function** onAdcInterrupt 7: // (2) When the ADC process is completed, ADC_interrupt is triggered. 8: // (3) Read ADC measurement results. 9: **igbt_temperature** = readAdc(temperature_channel) 10: **current** = readAdc(current_channel) 11: **voltage** = readAdc(voltage_channel) 12: // (3.1) Calculate optimal frequency based on inputs. 13: **freqHead** = calculateTargetFrequency(inputs) 14: // (3.2) Compare freqTail with freqHead. 15: **if** freqHead ≠ freqTail **then** 16: **frequencyDifference** = freqHead − freqTail 17: **if** |**frequencyDifference**|> freqDfDt **then** 18: // Update within frequency change limit. 19: **if** frequencyDifference > 0 **then** 20: **freqTail** = freqTail + freqDfDt 21: **else** 22: **freqTail** = freqTail − freqDfDt 23: **end if** 24: **else** 25: // Direct update if close enough. 26: **freqTail** = freqHead 27: **end if** 28: **fsw** = freqTail 29: **samplingFrequency** = calculateSamplingFrequency() 30: writeToPwmRegister(fsw) 31: writeToSamplingRegister(samplingFrequency) 32: **else** 33: // No update needed. 34: **do nothing** 35: **end if** 36: // Wait for next PWM period. 37: waitForNextPwmPeriod() 38: **end function**

## Appendix B. MLP-Based Assignment of Optimal Switching Frequency

**Algorithm A2** MLP-Based Assignment of Optimal Switching Frequency
1: **function** calculateTargetFrequency(current, duty, temp) 2: **Input:** motor_current, duty_cycle, temperature, weights, biases 3: **mlp_model** = Equation (15) 4: **optimum_frequency** = mlp_model.predict (motor_current, duty_cycle, temperature) 5: **current_frequency** = get_current_frequency() 6: **if** optimum_frequency == current_frequency **then** 7: **do nothing** 8: **else** 9: set_frequency(optimum_frequency) 10: **end if** 11: **end function**

## Appendix C. Locomotive Aerodynamic Equations

The aerodynamic equations of vehicles are used to calculate the aerodynamic forces, such as air resistance, encountered during motion. Vehicle aerodynamics is influenced by several factors, including speed, geometry, air density, and aerodynamic drag coefficient. Based on these factors, the required traction power is given by [51]:

(A1) $$\begin{array}{cc} P_{\mathrm{out}}=\; & P_{o}+\frac{1}{\eta_{m}}\left[\frac{1}{2}\rho C_{\omega }\left(\delta \right)H_{\mathrm{eff}}^{2}+m_{c}g\left(\sin\left(\alpha \right)+nc_{r2}\right)+m_{c}gc_{r1}\right]H_{\mathrm{avg}}. \end{array}$$

where:

- $P_{\mathrm{out}}$: Output power; net power used to move the vehicle.
- $P_{o}$: External power input; possibly supplied by the engine or other energy source.
- $\eta_{m}$: Motor efficiency coefficient.
- ρ: Air density (kg/m³).
- $C_{\omega }\left(\delta \right)$: Aerodynamic drag coefficient (depends on vehicle design and speed).
- $H_{\mathrm{eff}}$: Effective velocity, i.e., the airflow velocity around the vehicle.
- $m_{c}$: Vehicle mass (kg).
- *g*: Gravitational acceleration (9.81 m/s²).
- α: Road slope angle (radians).
- *n*: Number of wheels.
- $c_{r1}$, $c_{r2}$: Rolling resistance coefficients.
- $H_{\mathrm{avg}}$: Average velocity of the locomotive (m/s).

Equation (A1) defines the total power output of the vehicle on the left-hand side. On the right-hand side, it includes motor efficiency and resistance forces: aerodynamic drag, rolling resistance, and slope-induced forces. Based on this total output power, the corresponding motor current, the PWM duty cycle, and the motor load are calculated using the following expressions.

(A2) $$H_{\mathrm{avg}}=\frac{H_{\mathrm{init}}+H_{\mathrm{final}}}{2},$$

(A3) $$I_{\mathrm{motor}}=\frac{P_{\mathrm{out}}}{V_{\mathrm{nom}\_\mathrm{motor}}},$$

(A4) $$\%{\mathrm{Load}}_{\mathrm{motor}}=\frac{I_{\mathrm{motor}}\cdot 100}{I_{\mathrm{nom}\_\mathrm{motor}}},$$

(A5) $${\mathrm{RPM}}_{\mathrm{motor}}=\frac{H_{\mathrm{avg}}\cdot 60\cdot \mathrm{Gear}\;\mathrm{Ratio}}{\left(1-s\right)\cdot \pi \cdot R_{\mathrm{wheel}}},$$

(A6) $$V_{\mathrm{motor}\_\mathrm{applied}}=\frac{{\mathrm{RPM}}_{\mathrm{motor}}}{k_{n}},$$

(A7) $$\%\mathrm{Duty}=\frac{V_{\mathrm{motor}\_\mathrm{applied}}\cdot D_{\max}}{V_{\mathrm{nom}\_\mathrm{motor}}}.$$

where:

- $H_{\mathrm{init}}$, $H_{\mathrm{final}}$: Initial and final speeds of the locomotive (m/s).
- $I_{\mathrm{motor}}$: Motor current (A).
- $V_{\mathrm{nom}\_\mathrm{motor}}$: Nominal voltage of the motor (V).
- $I_{\mathrm{nom}\_\mathrm{motor}}$: Nominal current of the motor at full load (A).
- Gear Ratio: Gear ratio between the motor shaft and the wheel.
- *s*: Motor slip ratio.
- $R_{\mathrm{wheel}}$: Radius of the locomotive wheel (m).
- ${\mathrm{RPM}}_{\mathrm{motor}}$: Revolutions per minute of the motor.
- $k_{n}$: Motor speed fixed (RPM per Volt).
- $V_{\mathrm{motor}\_\mathrm{applied}}$: Voltage applied to the motor (V).
- %Duty: PWM duty cycle (%).
- $D_{\max}$: Maximum applicable PWM percentage.
